# Functional clustering of mouse ultrasonic vocalization data

**DOI:** 10.1371/journal.pone.0196834

**Published:** 2018-05-09

**Authors:** Xiaoling Dou, Shingo Shirahata, Hiroki Sugimoto

**Affiliations:** 1 Faculty of Science and Engineering, Waseda University, Tokyo, Japan; 2 Graduate School of Engineering Science, Osaka University, Osaka, Japan; 3 Division of Biology, Jichi Medical University, Shimotsuke, Tochigi, Japan; Universidade do Estado do Rio de Janeiro, BRAZIL

## Abstract

Mouse ultrasonic vocalizations (USVs) are studied in many fields of science. However, various noise and varied USV patterns in observed signals make complete automatic analysis difficult. We improve several methods to reduce noise, detect USV calls and automatically cluster USV calls. After reduction of noise and detection of USV calls, we consider USV calls as functional data and characterize them as USV functions with B-spline basis functions. For discontinuous USV calls, breakpoints in the USV functions are defined using multiple knots in the construction of the B-spline basis functions, and a hierarchical method is used to cluster the USV functions by shape. We finally show the performance of the proposed methods with USV data recorded for laboratory mice.

## Introduction

Mice are known to emit ultrasonic vocalizations (USVs) in many types of social behaviors, and the USVs seem to differ depending on a variety of contextual determinants; e.g., the age, genetic background and behavioral state affect the vocalization rate, syntax, frequency and duration [1]. This suggests that the USVs of mice convey information in social communication and this makes them important ([2], [3], [4], [5], [6], [7], [8]). It is also known that mice USVs contain different USV call (or syllable) types ([1], [9], [10], [11]). The importance of different patterns of USV calls has recently been reported; e.g., USVs emitted by males were shown to attract females in a playback test. It has been shown that female mice preferentially approach specific types of USVs emitted by males [12] and tend to approach USVs of a type that is emitted by a strain different from their own parents’ strain [13]. However, potentially important factors (e.g., the number of specific USV call types and the specific sequential emission pattern of call types) within different patterns of USVs used for social communication are still unclear. Clarifying differences among USV call types would help address this issue. The detailed characterization of USV call types requires many steps including recording, call detection, call type classification and clustering. These steps are taken using commercially available software. In these steps, call type classification and clustering are still time-consuming because noise reduction and pattern classification are done manually in most cases ([10], [12]). Currently available software (e.g., Avisoft Bioacoustics and the ISOMAP analysis method of [9]) can calculate and display spectrograms and allow automatic signal detection. However, suitable methods for the automatic classification and clustering of USV calls have not yet been developed.

We are interested in the variety of shapes of USV calls and we consider clustering call types. We take USV records from six laboratory male mice of two strains. The USV records contain not only USV phrases of syllables but also background noise, sounds of gnawing, squeaks (also called noisy syllables in [1]), movement noise and bedding noise of mice. USV calls appear in a frequency range higher than 30kHz and have several syllable types. Background noise can be considered uniformly distributed across all frequencies. The other sounds usually appear at frequencies lower than the frequencies of USV calls, but some spike into the range of USV frequencies. This was also pointed out by [9]. To focus on USV calls, we consider all other sounds as noise, estimate the weighted dominant frequency and detect USV calls.

To classify calls into subsets, [9] suggested considering each call as a function of time and clustering USV calls into several types by frequency jumps, but their analysis failed to cluster USV calls into subtypes. There was thus still a need for a new method of further subtype clustering. Similarly, [14] proposed a series of methods for handling continuous nonharmonic mouse USV data. The methods include procedures that reduce noise, find the signal of the calls and group continuous USV calls through functional clustering. Because USV calls are more often discontinuous in some datasets, we propose in this paper a method that employs B-spline basis functions to characterize USV calls as USV functions. Using this method, we can analyze both continuous and discontinuous nonharmonic USV calls. This overcomes the limitation of the orthogonal polynomial method introduced in [14] and reveals more subtypes of syllables with frequency jumps. We also make improvements to noise reduction; these can be considered a generalization of the method given in the same paper.

We consider nonharmonic USV call signals as functional data and represent the segments of frequencies as one-dimensional functions of time. Functional data analysis was proposed by [15, 16] and has been further developed and applied in many fields [17, 18]. Some functional clustering methods have been proposed for the unsupervised learning of functional data. As an example, [19] proposed a clustering method based on Gaussian mixture distributions for sparsely sampled functional data. [20] developed *k*-center functional clustering with functional principal component analysis. A two-stage clustering method was proposed by [21]; this method allows B-spline basis functions to be fitted to functional data and coefficient vectors to be partitioned through *k*-means clustering. We here propose a method similar to that in [21], using two-stage functional clustering with B-splines. However, our method is suitable for discontinuous curves. Furthermore, we apply Ward’s method for hierarchical clustering.

The paper is organized as follows. We first introduce our experiments and USV datasets. We then review the method of reducing noise and propose a weighted-frequency method to calculate frequency for each frame when detecting USV calls. After noise reduction, we define the USV calls as functions employing a B-spline method and cluster them employing a functional clustering method. We finally demonstrate our methods with six real USV datasets and present conclusions.

## Materials and methods

In experiments on testing mouse social behavior, we recorded USV data for three male mice of the strain BALB/cAnN and three male mice of the strain C57BL/6JJcl. The details of the experiments and the information of USV frames produced by fast Fourier transform of the data are given as follows.

### Experiments and datasets

Ethics statement

Mice were maintained in accordance with National Institute of Genetics (NIG) guidelines. This study was carried out in strict accordance with the recommendations in the Guidelines for Proper Conduct of Animal Experiments of the Science Council of Japan. The protocol was approved by the Institutional Committee for Animal Care and Use of the National Institute of Genetics (Permit Numbers: 21-14).

Animals

Ultrasound emissions from male mice C57BL/6JJcl were recorded during male–female interaction. C57BL/6JJcl were purchased from CLEA Japan, Inc. (Tokyo, Japan). Mice were bred at the NIG and used in the experiments. All animals were kept at the NIG under a 12-h light/dark cycle (light from 8:00 to 20:00) in a temperature-controlled room.

Apparatus

An ultrasound microphone (CM16/CMPA Condenser ultrasound microphone, Avisoft Bioacoustics) and recorder (UltraSoundGate 116H, Avisoft Bioacoustics) were used for recording. The microphone was positioned approximately 10 cm above a cage that contained the mice ([12]).

Recording procedure

A 15-week-old male C57BL/6JJcl was paired with a 15-week-old female C57BL/6JJcl for 1 week. Two days before the test day, the male mouse was housed individually. Another 15-week-old female C57BL/6JJcl was injected with pregnant mare serum gonadotropin to control its sexual cycle. On the test day, the male mouse was transferred to a small cage (12 cm ×20 cm) with wood chip bedding, and the second female mouse was then introduced into the small cage. Immediately after the female had been introduced, the recording of sound began. The recording was performed during the early part of the dark phase, 20:00–24:00 pm. Ultrasound data for BALB/cAnN were obtained from [12].

Datasets and fast Fourier transform

The six raw datasets recorded for the two mouse strains were transformed by fast Fourier transform into six sequences of USV frames as follows. Each frame had a duration of 0.85 ms. Each USV frame overlapped 50% with the next frame, and is represented by 256 samples taken at a sampling rate of 300kHz; i.e., the highest measurable frequency (the Nyquist frequency) was 150kHz, which is half the sampling frequency, and the time between adjacent frames was 0.425 ms. Hence for each frame, 126 squared amplitudes (or powers) were computed for the sampling frequencies. The durations and transformed matrix sizes of the datasets are listed in Table 1. Our analysis uses the obtained matrices.

**Table 1:** Durations and the transformed matrix sizes of the datasets.

Dataset	Duration(seconds)	Size of obtained matrix
S1 Data	99	233393 × 126
S2 Data	299	703182 × 126
S3 Data	299	703182 × 126
S4 Data	188	443086 × 126
S5 Data	320	753614 × 126
S6 Data	302	709966 × 126

Each dataset is expressed by a matrix *X* = {*x*_*i*,*j*_}, where *i* = 1, …, *T* and *j* = 1, …, *F* are for time and frequency, respectively. The entries, *x*_*i*,*j*_, are intensities corresponding to the level of vocalization.

### Noise reduction

A noise reduction process has been proposed for mouse USV data [14]. The process involves three steps: taking a moving average, determining the minimum USV call intensity and separating the USV call signals from the matrix. This subsection briefly reviews these three steps and then makes an improvement to the third step.

The first step is to use a small matrix of size *m* × *n* to calculate the two-dimensional moving average of the original matrix data. This gives the moving-average matrix

$$\begin{array}{c} \tilde{X}=\left\{{\tilde{x}}_{1},\cdots ,{\tilde{x}}_{T-m+1}\right\}=\left\{{\tilde{x}}_{k,l}\right\}, \end{array}$$

where

$$\begin{array}{c} {\tilde{x}}_{k,l}=\frac{1}{mn}\sum_{i=k}^{k+(m-1)}\;\sum_{j=l}^{l+(n-1)}x_{i,j},\;k=1,\cdots ,T-m+1,\;\;\;l=1,\cdots ,F-n+1, \end{array}$$

with *m* and *n* respectively indicating time and frequency. Moving averages are often used in time series to model the trend of data. The purpose of the moving-average step here is to reduce perturbation in the signals, which helps in distinguishing noise from the voice signal and finding the trend of the USV signals.

The second step is to choose a threshold intensity, according to the noise level, and to set all entries smaller than the threshold in the moving-average matrix $\tilde{X}=\left\{{\tilde{x}}_{k,l}\right\}$ to zero. This effectively reduces the background noise in the experiment. Low-frequency noise and noise spiking from low frequency to high frequency can be removed by setting the intensities at those frames to zero.

The third step is to detect USV calls. [14] chose the frequency corresponding to the maximum intensity for each frame ${\tilde{x}}_{i}$, *i* = 1, ⋯, *T* − *m* + 1. When a frame has more than one maximum, the median frequency among those with maximum intensity is chosen.

Choosing the frequency from the maximum intensity may result in insufficiently smoothed USV curves. We here propose using a weighted frequency as a generalization of using the maximum intensity. For time *i*, the weighted frequency can be defined by

$$\begin{array}{c} f_{i}=\sum_{j}\frac{x_{i,j}^{*}}{\sum_{k}x_{i,k}^{*}}j, \end{array}$$ (1)

where

$$\begin{array}{c} x_{i}^{*}=\text{quantile}\left({\tilde{x}}_{i},p\right)=\left(x_{i,j}^{*}\right) \end{array}$$

is a vector containing elements of ${\tilde{x}}_{i}$ larger than the 100*p*% quantile. We see that the frequency is thereby weighted by the ratio of $x_{i,j}^{*}$ in the largest 100(1 − *p*)% quantile intensities in ${\tilde{x}}_{i}$.

This produces a sequence of frequency in time. The sequence contains USV calls and interspaces of different durations. To detect the USV calls from the sequence, we need to determine the interspaces between adjacent USV calls. [14] proposed determining the length of interspaces by considering the total number of USV calls for different lengths of interspace. Choosing a threshold for interspacing that is too short may separate the sequence into too many segments; in contrast, choosing a threshold that is too long may merge distinct USV calls into a long segment. A plot of the total number of USV calls for different lengths of interspace can suggest the proper length of interspace, at which the total number of USV calls becomes stable. The obtained length of interspace, which we will call *l*, is the shortest length of the interspaces between adjacent frequency USV calls. That is to say, if two adjacent frequency points are separated by *l* or more units of time, we judge that they are from distinct USV calls; otherwise they are considered to be part of the same USV call.

From this, we obtain USV calls with different lengths. Since very short USV calls may be noise, we remove these short “USV calls” to conclude the noise reduction process.

### Definition of functional data

Our purpose is to classify the USV calls by shape. We consider the USV calls as functional data and define them as functions. Many methods are available for estimating USV functions, such as the use of Chebyshev polynomials [22], kernel methods, and wavelet methods. Because almost all USV calls from the BALB/cAnN mouse are continuous, [14] proposed the use of Chebyshev polynomials to model the USV calls of the BALB/cAnN mouse. However, this method cannot be applied to discontinuous USV calls.

Because many USV calls from the C57BL/6JJcl mice are not continuous, they may contain several jumps or breakpoints. We prefer the B-spline basis function method [23] for this reason. The B-spline basis function method is one of the most popular methods of representing data as functions. Libraries of code for working with B-spline functions are available in many programming languages, including R, S-Plus and MATLAB. In this paper, we use the function bs() in the splines package for R.

A B-spline basis function is a spline function defined by its order and a sequence of knots. Let

$$\begin{array}{c} \tau_{-d}=\cdots =\tau_{0}=0\leq \tau_{1}\leq \cdots \leq \tau_{m-1}\leq 1=\tau_{m}=\cdots =\tau_{m+d} \end{array}$$ (2)

be *m* + 2*d* + 1 knots on [0, 1]. We denote by *β*_*k*,*d*_(*t*) the *k*th B-spline basis function of order *d* for the knot sequence in (2). *β*_*k*,*d*_(*t*) is then defined recursively in terms of divided differences:

$$\begin{array}{ll} \beta_{k,d}\left(t\right) & =\frac{t-\tau_{k}}{\tau_{k+d}-\tau_{k}}\beta_{k,d-1}\left(t\right)+\frac{\tau_{k+d+1}-t}{\tau_{k+d+1}-\tau_{k+1}}\beta_{k+1,d-1}\left(t\right),\\ & k=-d,\cdots ,-1,0,1,\cdots ,m-1 \end{array}$$

with the initial condition

$$\begin{array}{c} \beta_{k,0}\left(t\right)=\{\begin{array}{cc} \frac{1}{\tau_{k+1}-\tau_{k}} & \tau_{k+1}>\tau_{k}\;\;\text{and}\;\;t\in \left[\tau_{k},\tau_{k+1}\right),\\ 0 & \text{otherwise}. \end{array} \end{array}$$

This process generates *P*(≔ *d* + *m*) B-spline basis functions. We know that any linear combination of a set of B-spline basis functions is still a spline function that is a piecewise polynomial in which the adjacent polynomials join smoothly at the knots. An essential property of B-splines is that knot duplication reduces continuity. If we duplicate an interior knot in the construction of the knot sequence, and generate the B-spline sequence, then the linear combination of B-splines has one less continuous derivative at the duplicated knot. This means that constructing functions with breakpoints is straightforward.

Assume that we have *N* USV calls. There are *n*_*i*_ pairs of (*t*_*i*,*j*_, *f*_*i*,*j*_), *j* = 1, ⋯, *n*_*i*_, in the *i*th USV call, *i* = 1, ⋯, *N*. To define a spline, we first specify the degree of the B-splines and knots over the interval where the function is to be approximated. These choices determine the goodness of fit of the functions. Cubic splines (*d* = 3) and quantiles of $\left[t_{i,1},t_{i,n_{i}}\right]$ are typical choices for piecewise polynomials and interior knots, respectively. Without getting deeply involved in model selection, we suggest setting single interior knots at the same quantiles for both continuous and discontinuous USV calls. For discontinuous USV calls, we specify a constant *κ* as the threshold. When |*f*_*i*,*j* − 1_ − *f*_*i*,*j*_| > *κ*, the curve is considered discontinuous at time *j*, and *j* is called a breakpoint. To construct a jump at a breakpoint in the regression function, we put *d* + 1 knots at the breakpoint. Here, *d* is the degree of the B-spline functions. This allows us to obtain the same length of coefficient vectors for the USV calls with the same number of breakpoints.

For all USV calls, we assume that

$$\begin{array}{c} f_{i,j}=\theta_{i,0}+\sum_{p=1}^{P}\theta_{i,p}\beta_{p,d}\left(t_{i,j}\right)+\varepsilon_{i,j};\;p=k+d+1;\;\;k=-d,\cdots ,0,\cdots ,m-1;\;\;P=d+m \end{array}$$

or in matrix form,

$$\begin{array}{c} f_{i}=B\theta_{i}+\varepsilon_{i}, \end{array}$$

where $t_{i}={\left(t_{i,1},\cdots ,t_{i,n_{j}}\right)}^{\prime }$, $f_{i}={\left(f_{i,1},\cdots ,f_{i,n_{j}}\right)}^{\prime }$ respectively indicate time and frequency. $B=(1,\beta_{1,d}\left(t\right),\cdots ,\beta_{P,d}\left(t\right))$ is then the set of B-spline basis functions with degree *d*. Using the least-squares method, for each USV call *i*, we obtain the regression function

$$\begin{array}{c} {\hat{f}}_{i}=B{\hat{\theta }}_{i}, \end{array}$$

with coefficient vector

$$\begin{array}{c} {\hat{\theta }}_{i}={\left(B^{\prime }B\right)}^{-1}B^{\prime }f_{i}. \end{array}$$

Hence, for each curve, we obtain a coefficient vector of length *P* + 1 = *d* + *m* + 1.

### Functional clustering

We propose a two-step clustering method for splitting the obtained USV curves into clusters. In the first step, we group the curves by the number of breakpoints. Curves with the same number of breakpoints have the same length of coefficient vectors and should be gathered into the same cluster. These clusters are called the first-level clusters, or groups.

In the second step, we separate the curves in each first-level cluster by shape. This process is performed by clustering the coefficient vectors, using standard multivariate clustering methods, such as Ward’s method.

## Results

### Analysis of BALB/cAnN data

**Fig 1:**
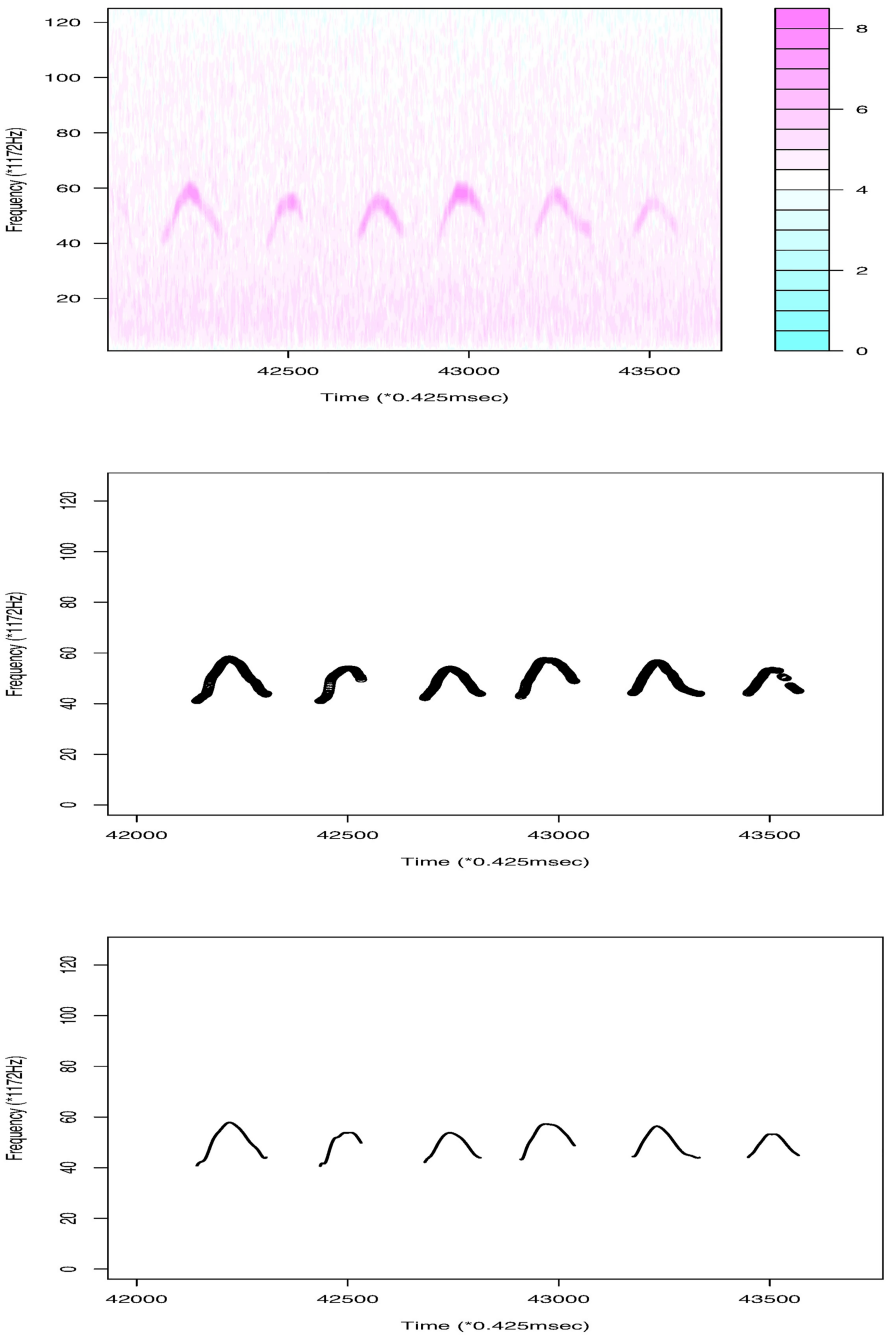
The first panel shows a part of the original data (log transformed). After the moving average is taken and background noise is reduced, we obtain the sequence of USV signals using weighted frequencies in the second panel and the USV functions generated by the B-spline method in the last panel.

**Fig 2:**
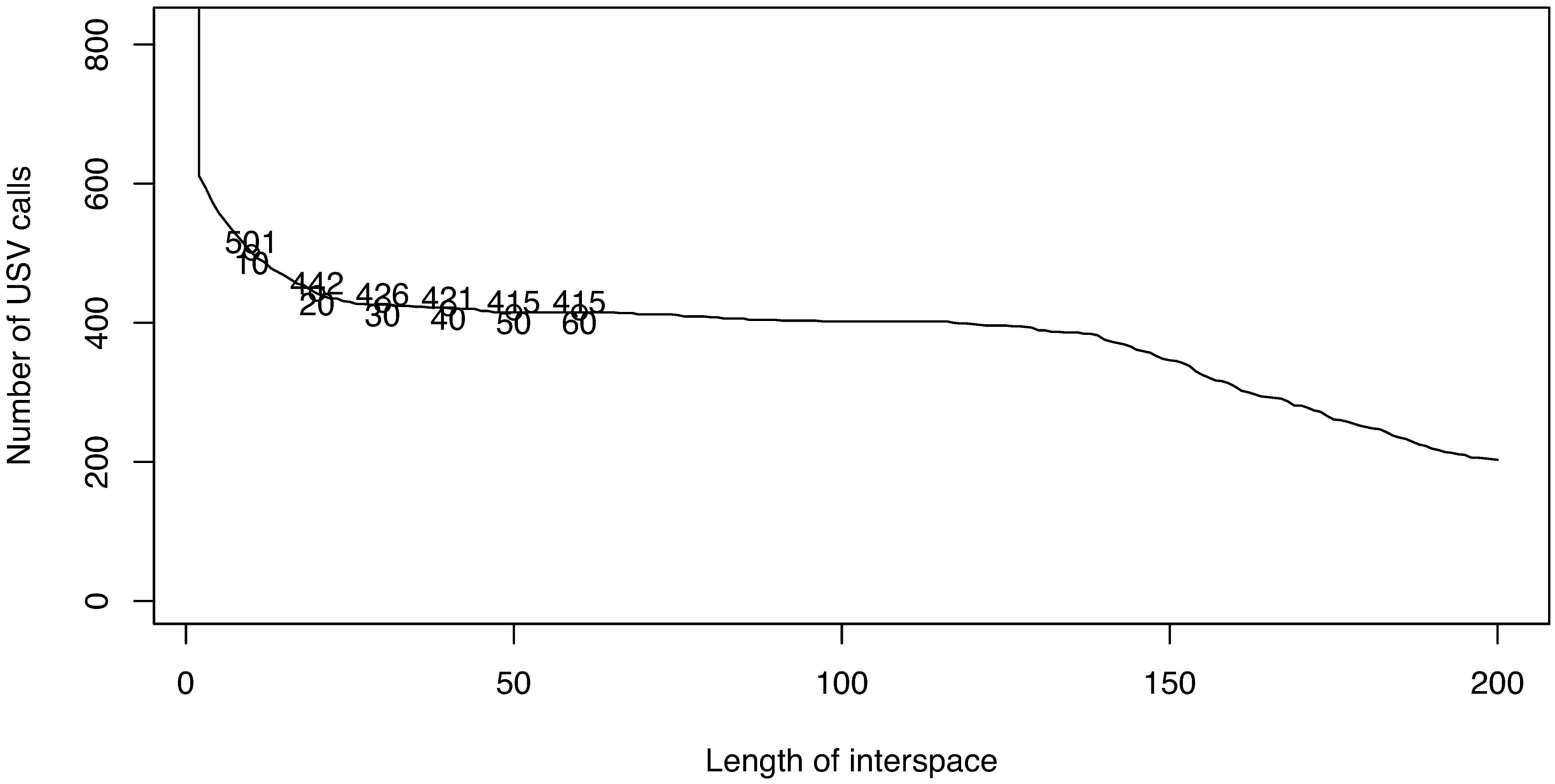
The shortest length of interval between adjacent USV calls can be determined from the change in the total number of USV calls while adjusting the length.

Our first example is a dataset (S1 Data) from a BALB/cAnN male mouse, BALB/cAnN 1752. The intensity level range is [5, 5330]. To reduce noise, we first apply the moving-average method with a small matrix of size 15 × 3. We then set intensities less than 200 to 0. Because almost all USV calls are at a frequency larger than 40 × 1172Hz, signals in this range are considered. By considering the largest 5% quantile intensities at each frame, a sequence of weighted frequency (1) over time is obtained. Fig 1 shows part of the original data and the results of the noise reduction method in the first two panels. Fig 2 shows that the total number of USV calls becomes stable when the length of the interspace between two adjacent USV calls is 30. We thus set 30 × 0.425 ms as the shortest length possible between adjacent USV calls. After deleting 33 short “USV calls” (shorter than 15 × 0.425 ms = 6.375 ms), we obtain 393 USV calls for analysis. Fig 2 reproduces the result obtained using the maximum-frequency method in [14]. The figure shows that the weighted frequency method achieves the same result as the maximum frequency method and can be considered a generalization of the maximum frequency method.

**Fig 3:**
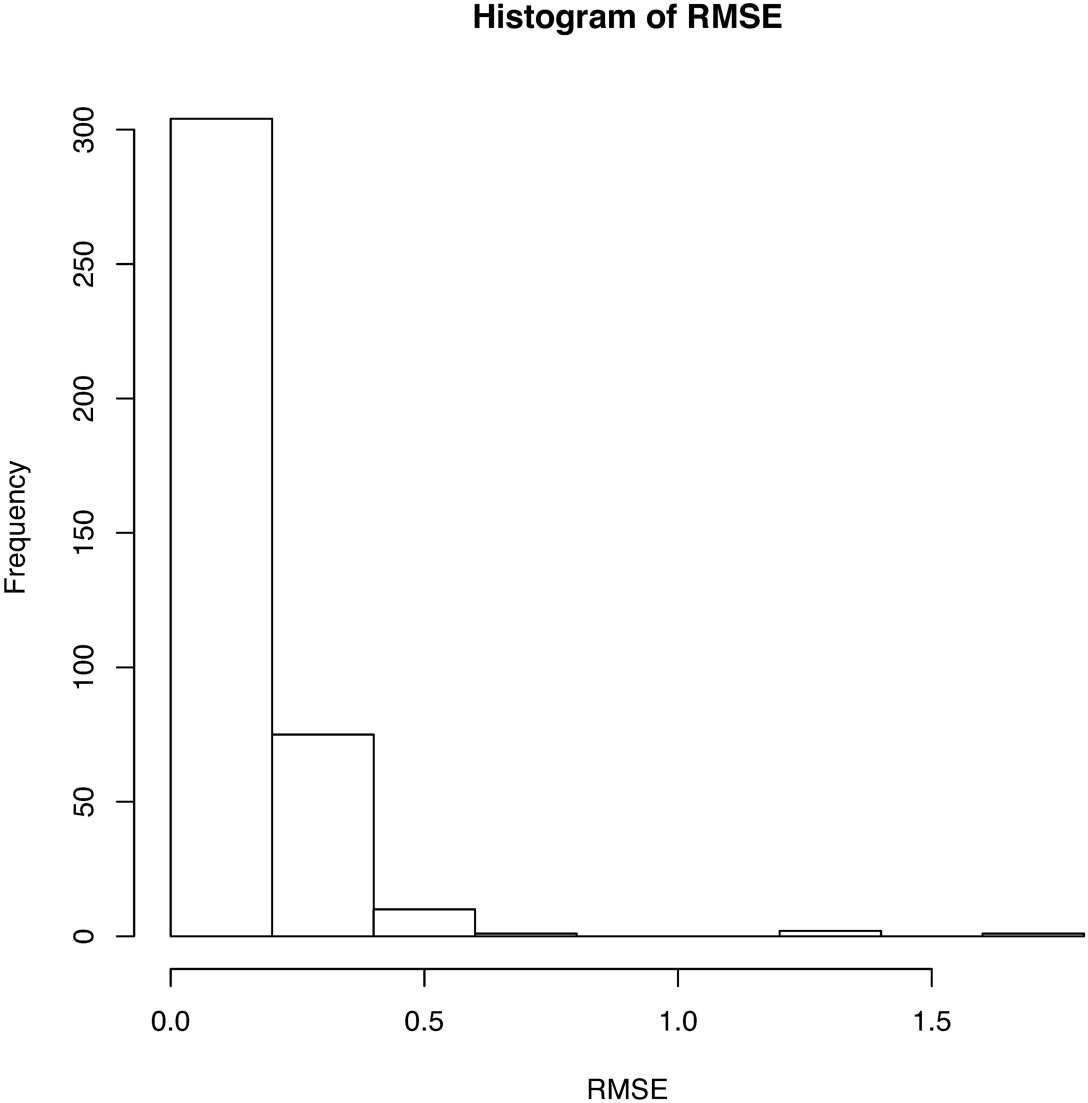
Histogram of RMSEs for fitting 393 USV curves. The mean of the RMSEs is 0.143, while the variance is 0.024.

We regard the obtained signals of USV calls as functional data and use the B-spline regression to define them as functions. Because almost all USV patterns are continuous, we set eight equally spaced interior knots on the domain of each USV curve. To define breakpoints, we set *κ* = 8. We then add four multiple knots for each breakpoint and fit the signals with cubic B-spline basis functions. From this, we finally obtain 386 continuous USV functions, six USV functions with one breakpoint, and one USV function with two breakpoints. The obtained functions for the USV call examples are given in the last panel of Fig 1. A histogram of the roots of mean squared errors (RMSEs) for the 393 USV curves is presented in Fig 3. The mean and variance of the RMSEs are respectively 0.143 and 0.024.

The final step is functional clustering. The 393 functions are first classified by their number of break points into three groups: continuous curves (Group I), discontinuous curves with one breakpoint (Group II) and discontinuous curves with two breakpoints (Group III).

**Fig 4:**
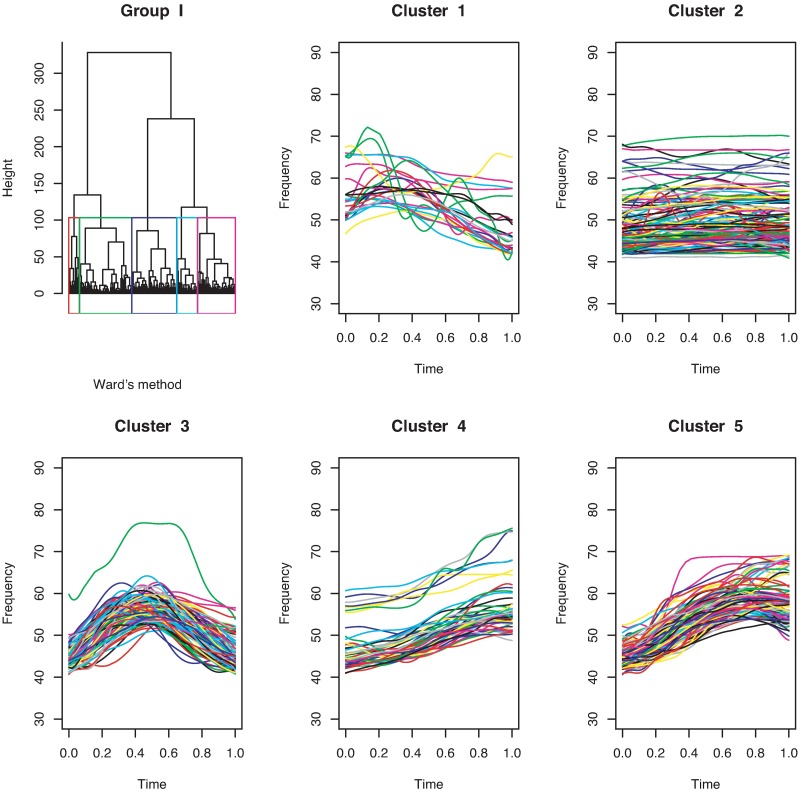
Cluster dendrogram and clusters obtained by clustering the coefficient matrix of continuous USV functions for mouse BALB/cAnN 1752 using Ward’s method.

**Fig 5:**
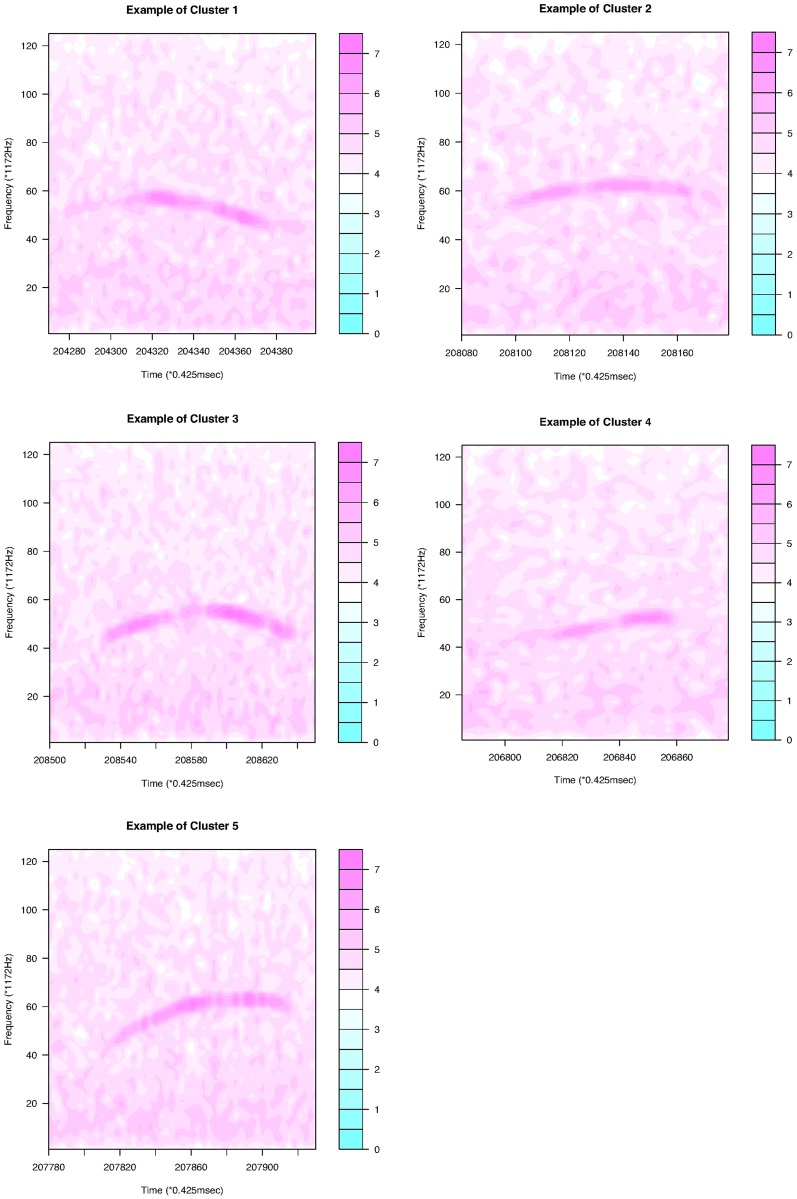
Examples of the five clusters in Group I.

**Fig 6:**
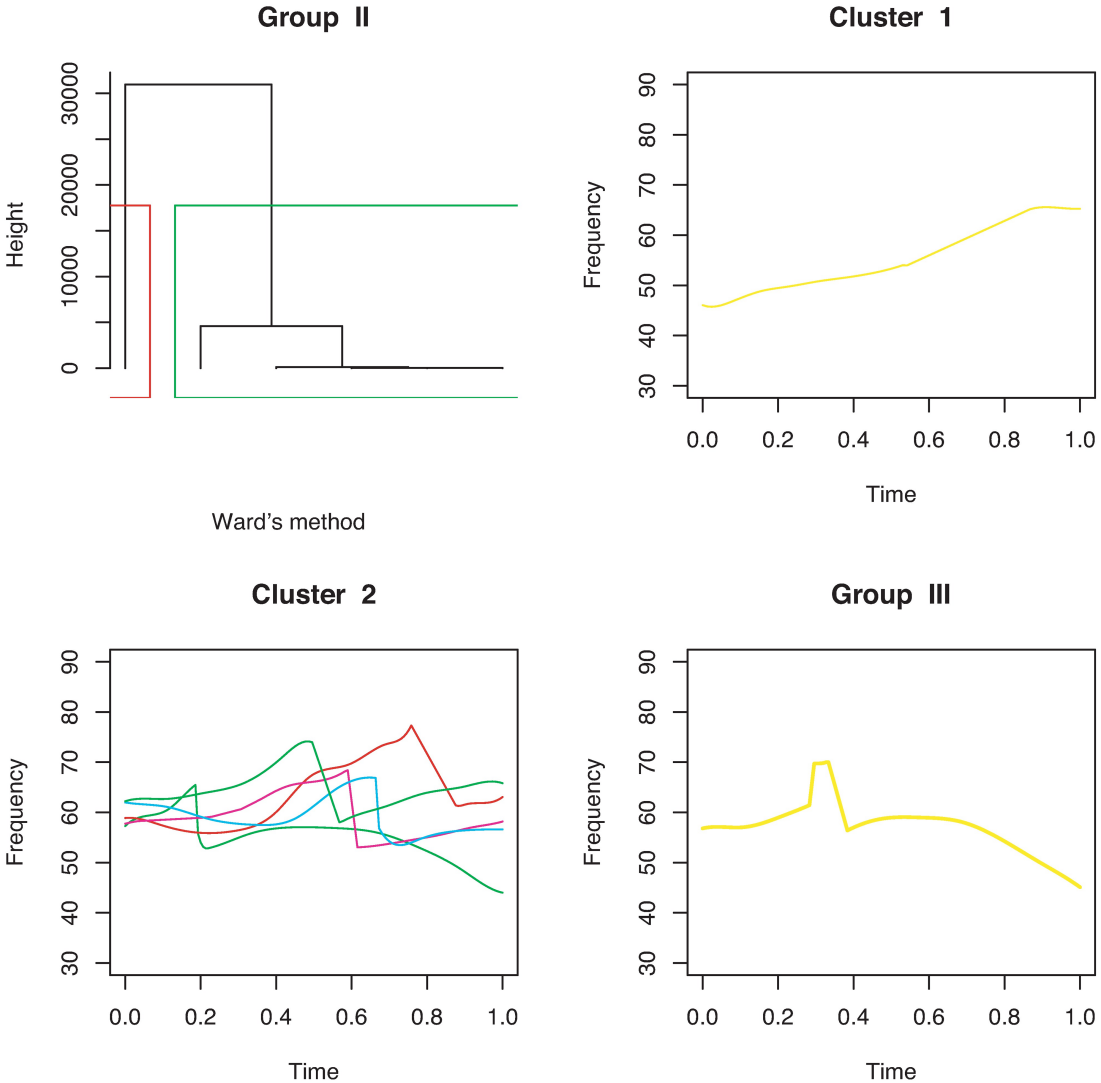
Cluster dendrogram and clustering of discontinuous USV functions with one breakpoint for mouse BALB/cAnN 1752 are shown in the first three panels. The USV function with two breakpoints is shown in the last panel.

**Fig 7:**
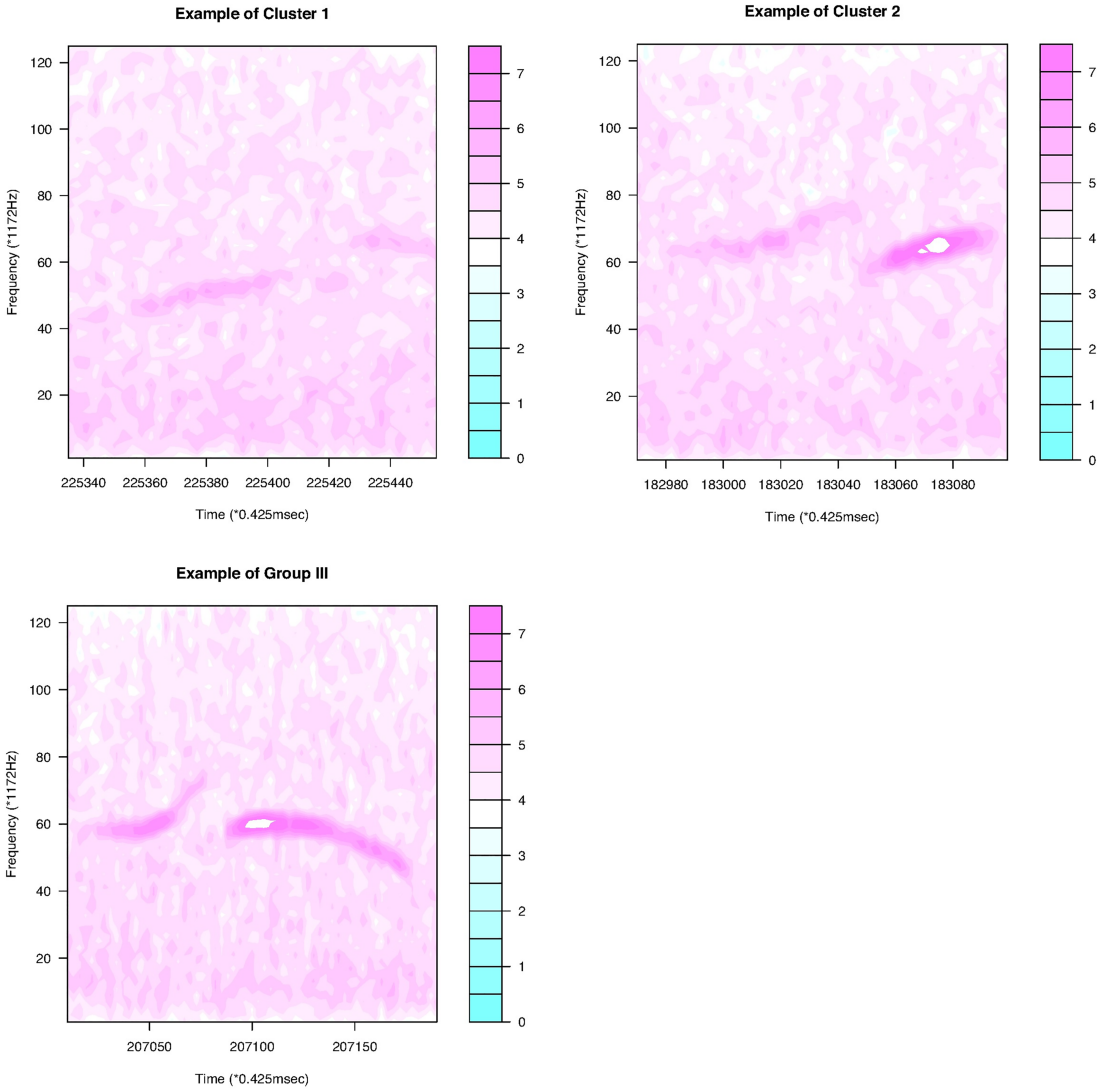
Examples of the two clusters in Group II and the USV call in Group III.

For continuous curves, we use Ward’s [24] clustering criterion to obtain a dendrogram, as shown in Fig 4. We can divide the 386 continuous curves in Group I into three or five clusters. Because we consider only the shapes of the curves, all the curves are plotted in the interval of time [0, 1]. In the case of splitting Group I into five clusters, we have five types of syllable shapes: downward, flat, mound, upward, and mound-upward (see examples in Fig 5). Group II can be divided into two clusters as jump-up and jump-down shapes (Fig 6). Only one discontinuous USV function has two breakpoints in Group III; this is shown in the last panel of Fig 6. Examples for Groups II and III are given in Fig 7.

USV datasets from the other two BALB/cAnN mice are analyzed in the same way, except that the threshold intensity is chosen as 130 because of the low intensity ranges of the datasets. From data of mouse BALB/cAnN 1563, we detected 25 USV calls, among which 24 are continuous and one is discontinuous. The calls are clustered in S1 File of Supporting information. From the data of mouse BALB/cAnN 1565, we obtain 189 continuous USV calls and four discontinuous USV calls. The clustering is given in S2 File of Supporting information.

### Analysis of C57BL/6JJcl data

**Fig 8:**
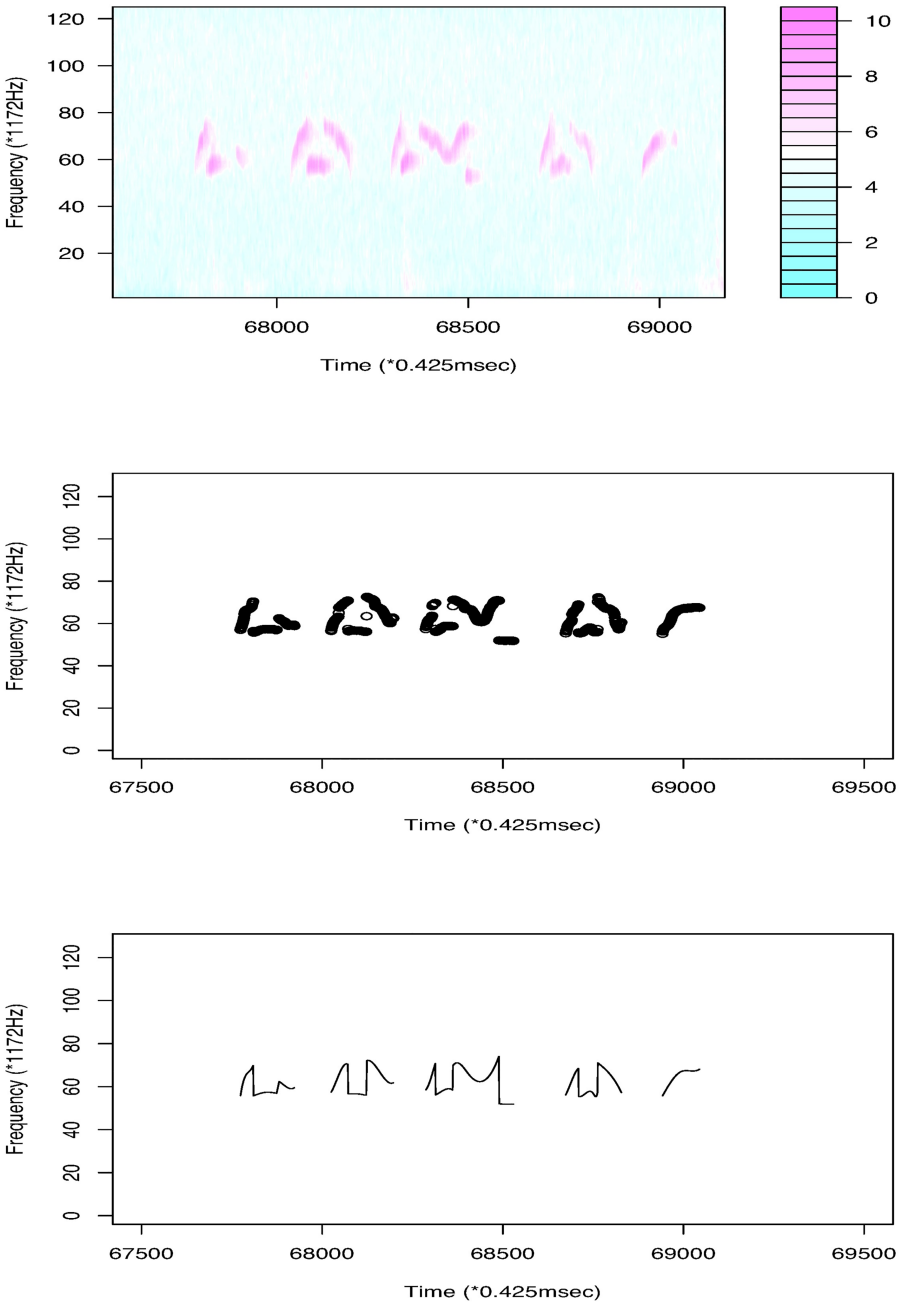
The first panel shows some of the data (log transformed) for mouse C57BL/6JJcl 2358. The second panel is the result after noise reduction. The last panel shows the USV curves obtained using the B-spline basis function method.

**Fig 9:**
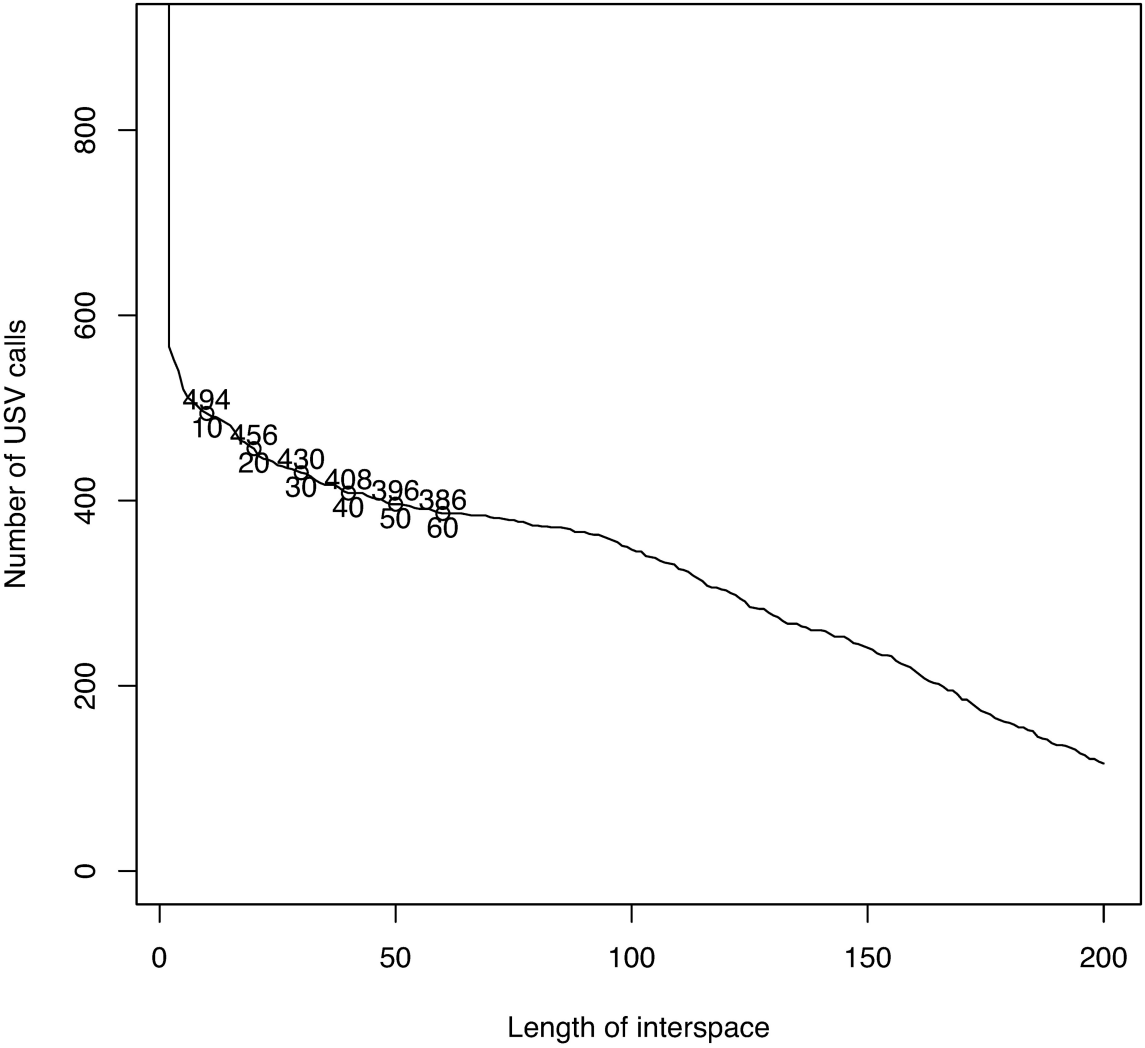
Determining the interval length between adjacent USV calls.

**Fig 10:**
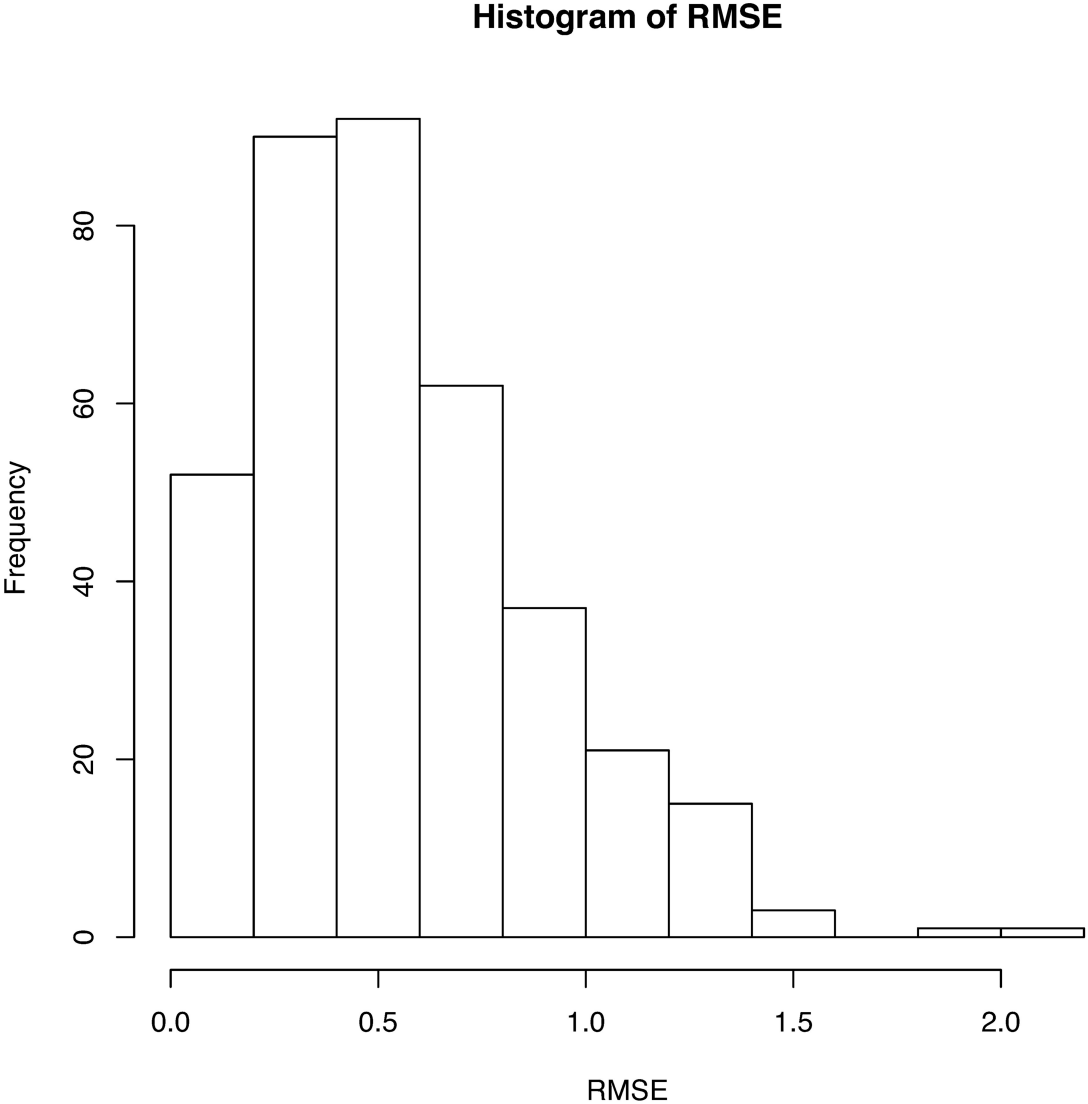
Histogram of RMSEs for the fitting of 374 USV curves. The mean of the RMSEs is 0.546 and the variance is 0.118.

This subsection makes a detailed analysis with a dataset (S4 Data) for a C57BL/6JJcl male mouse, C57BL/6JJcl 2358. Different strains of mice produce different types of USV calls. The first panel of Fig 8 shows some USV calls of this mouse. We see that some USV curves are continuous; however, many USV curves contain jumps. Considering the intensity level ([0, 32766]), we choose the largest 2% quantile intensities at each frame and obtain 430 USV calls, as seen in Fig 9. For this dataset, we put only one interior knot at the middle of the interval for each curve, and set *κ* = 5 as the threshold with which to define breakpoints. Using the same method as the first dataset, we reduce the noise and delete 56 short “USV calls.” Finally, 374 USV functional data items are used. Fig 10 presents a histogram of RMSEs of fitting. The mean and variance of the RMSEs are respectively 0.546 and 0.118. According to the number of breakpoints, we divide all USV calls into five groups (Table 2). The curves in the first four groups are then separated further by shape. Cluster dendrograms and clusters are obtained by clustering the coefficient matrix of USV functions of each group using Ward’s method.

**Table 2:** Three-hundred and seventy-four USV curves are divided into five groups by the number of breakpoints.

Group	I	II	III	IV	V
Number of breakpoints	0	1	2	3	more than 3
Number of USV curves	115	95	50	42	72

**Fig 11:**
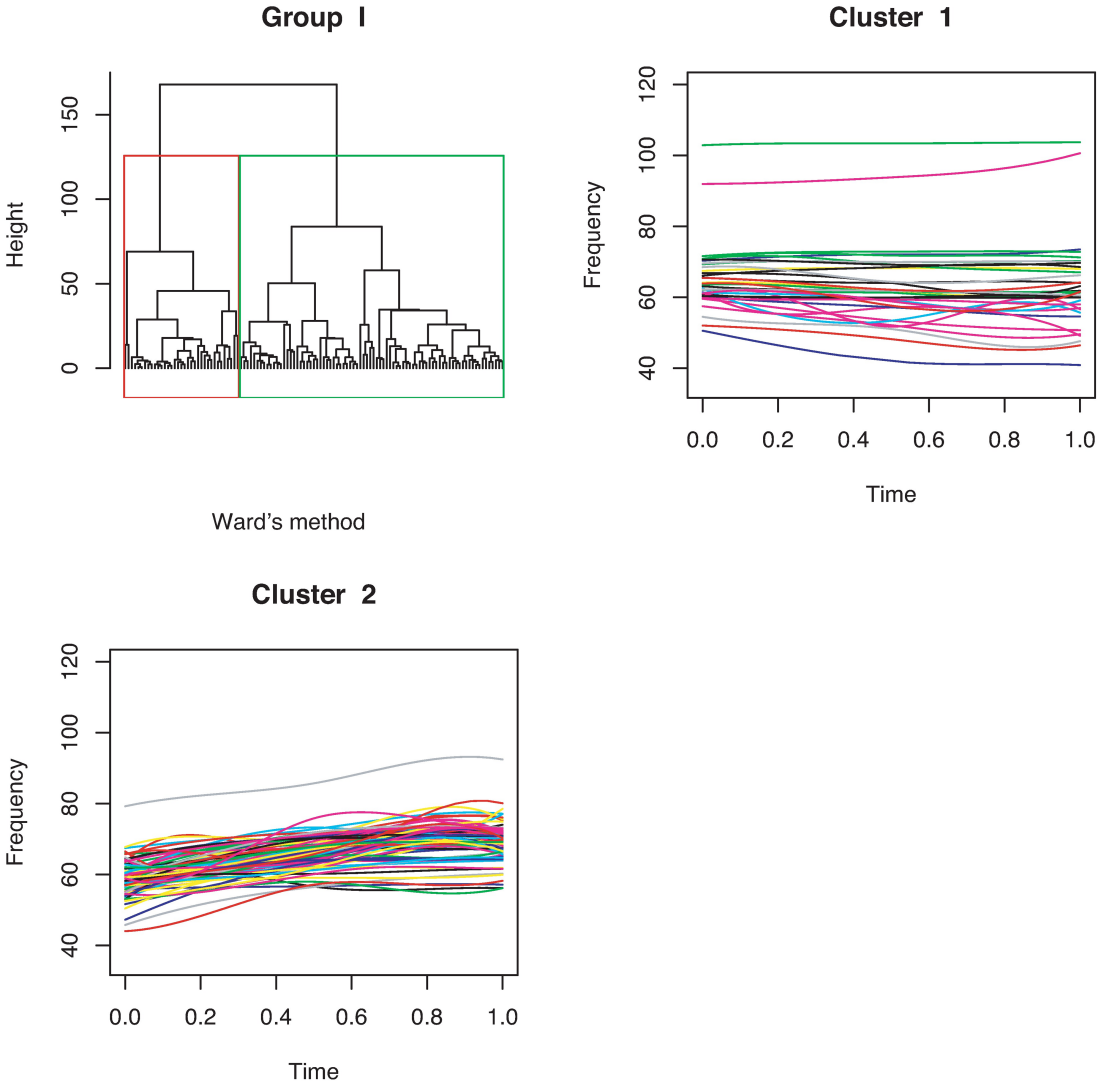
Cluster dendrogram obtained for continuous functions and two clusters.

**Fig 12:**
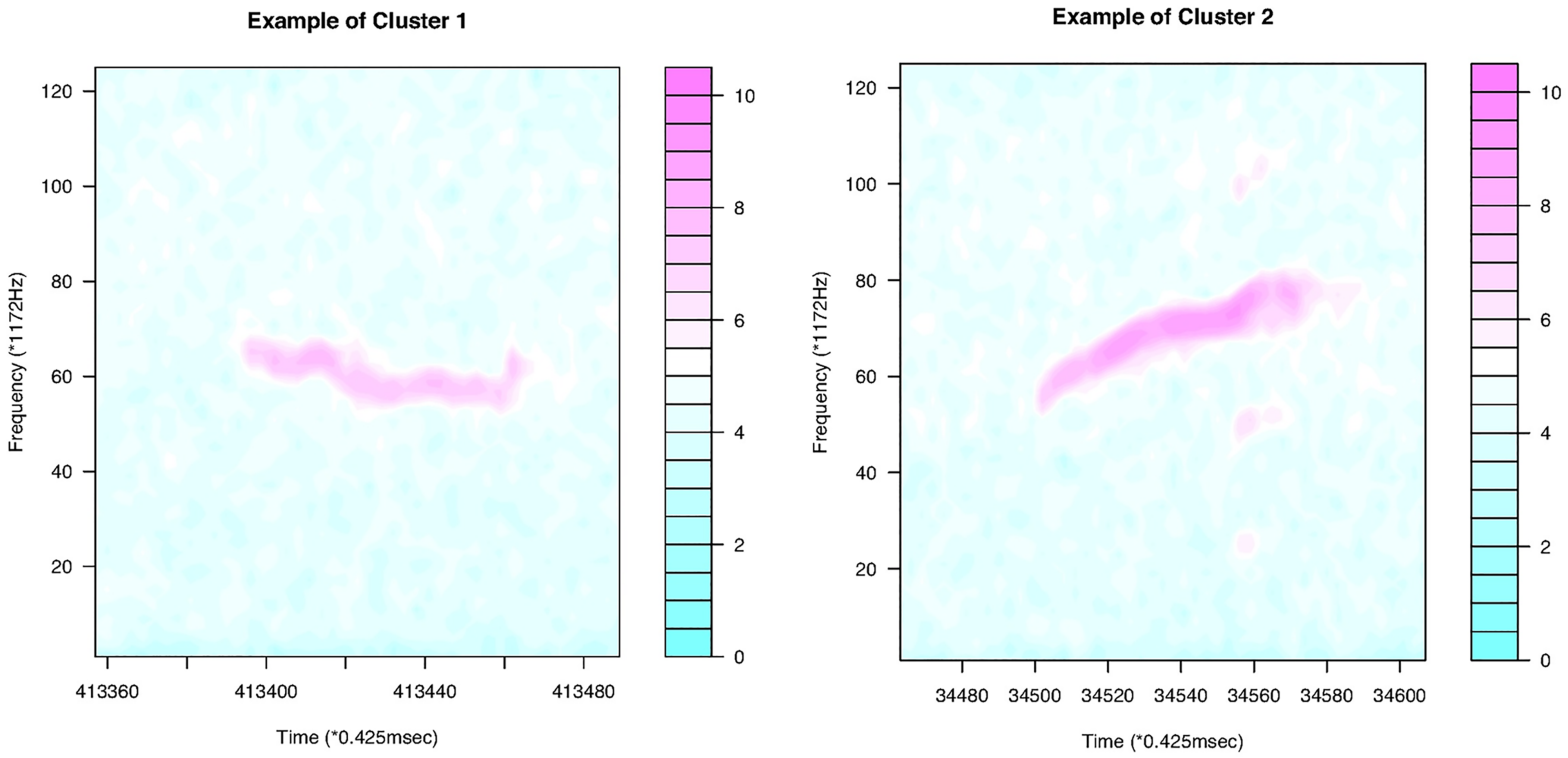
Examples of the two clusters in Group I.

**Fig 13:**
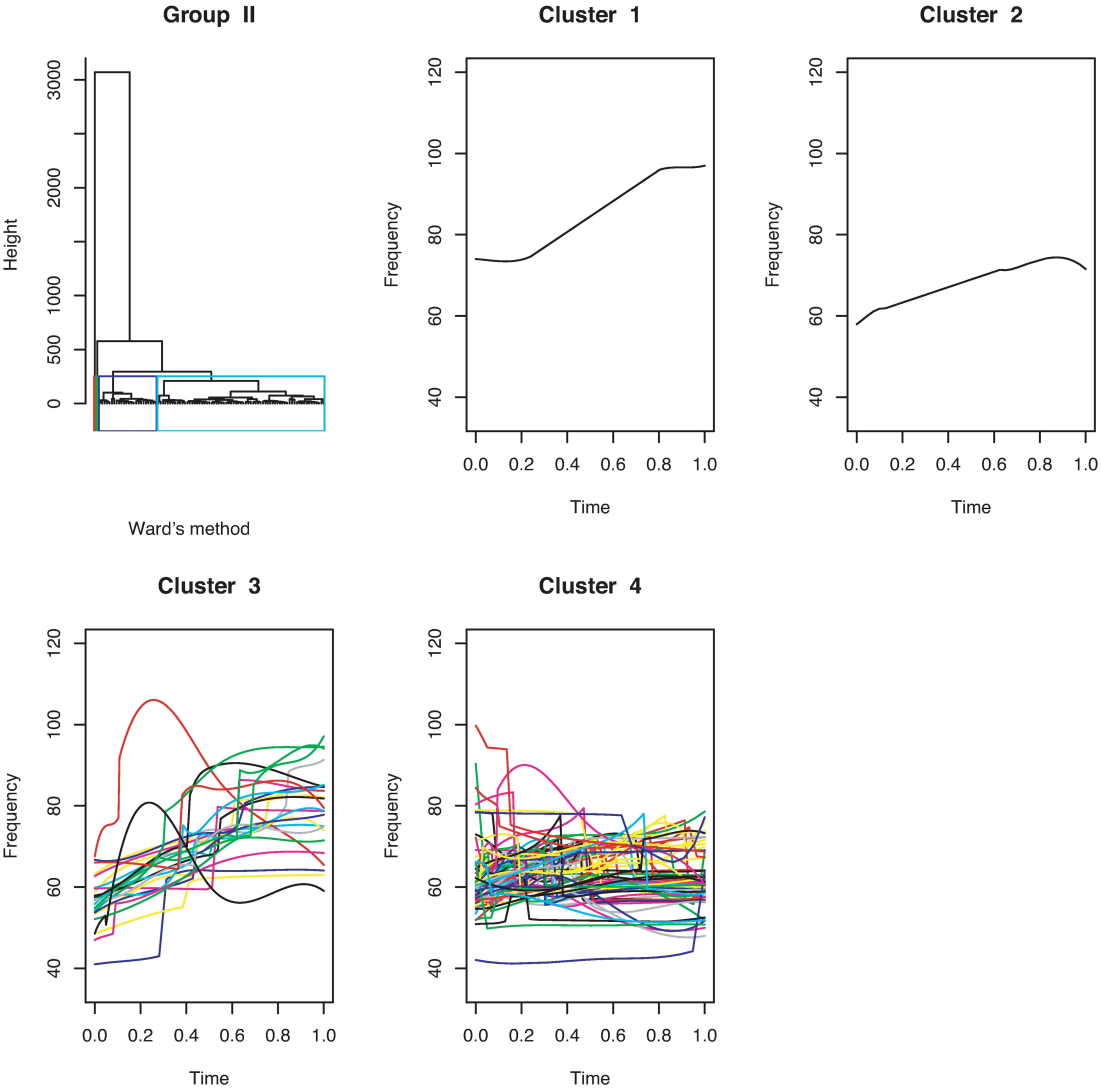
Clustering of discontinuous USV functions with one breakpoint for mouse C57BL/6JJcl 2358.

**Fig 14:**
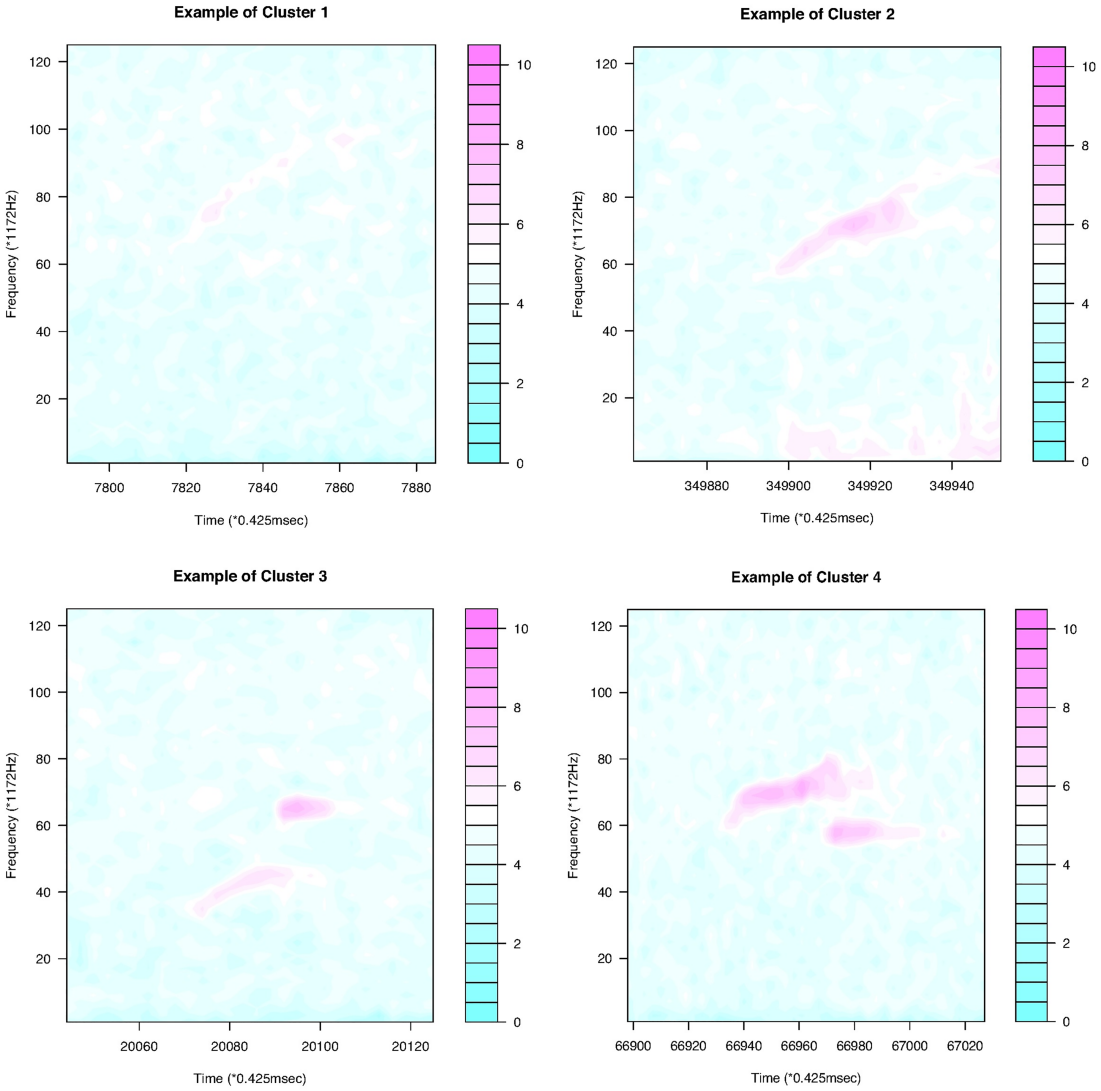
Examples of the four clusters in Group II.

**Fig 15:**
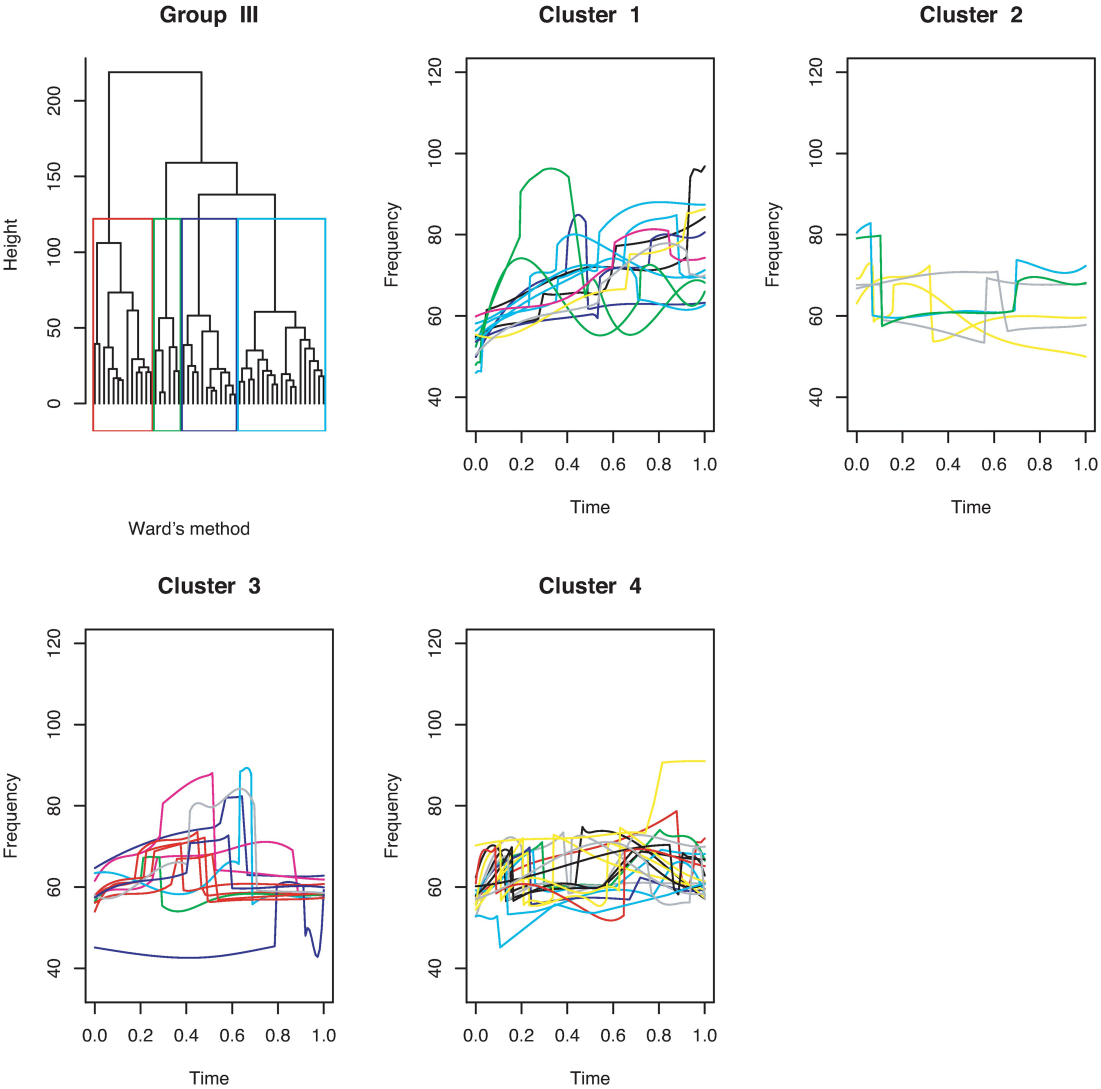
Clustering of discontinuous USV functions with two breakpoints for mouse C57BL/6JJcl 2358.

**Fig 16:**
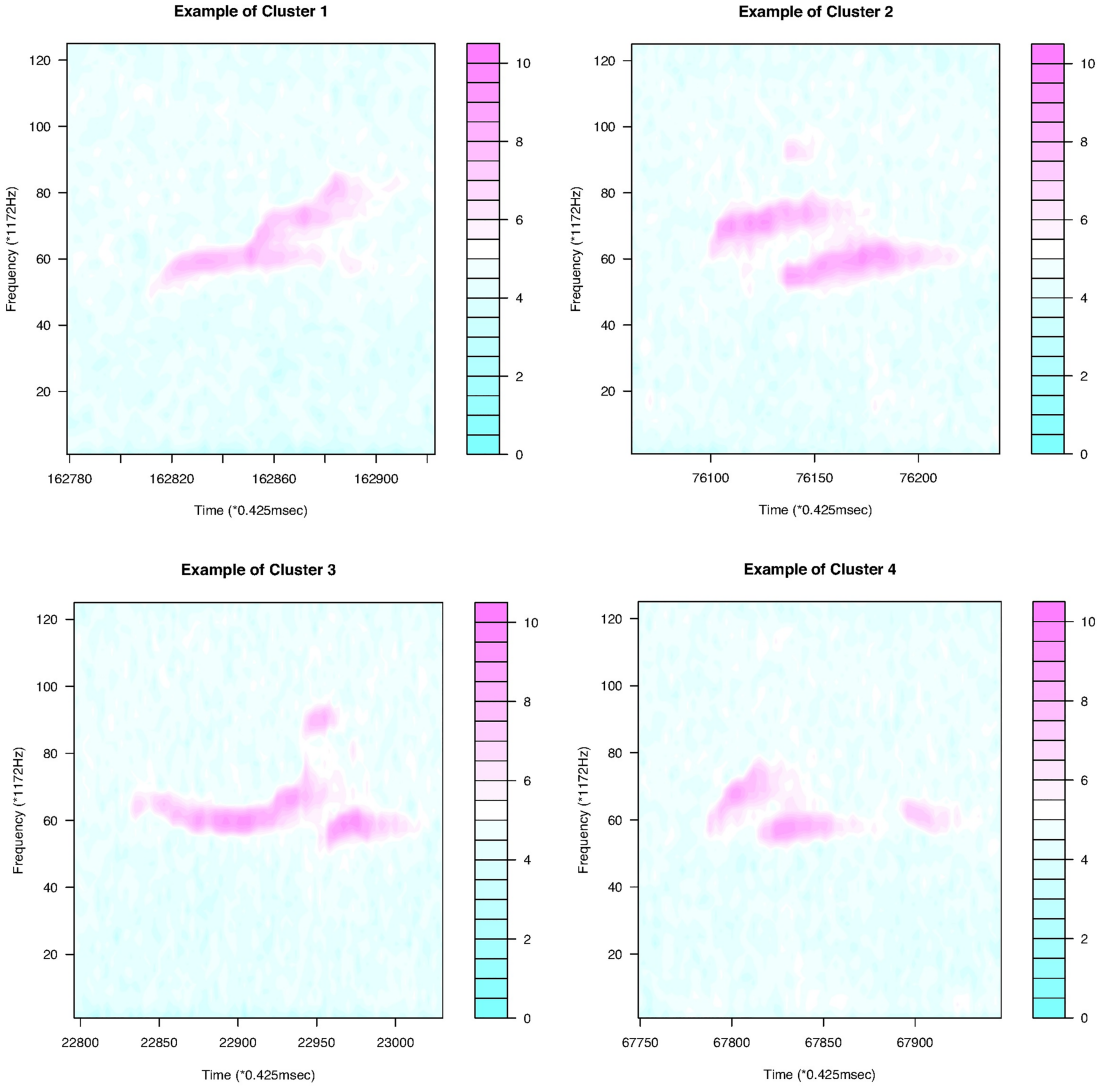
Examples of the four clusters in Group III.

**Fig 17:**
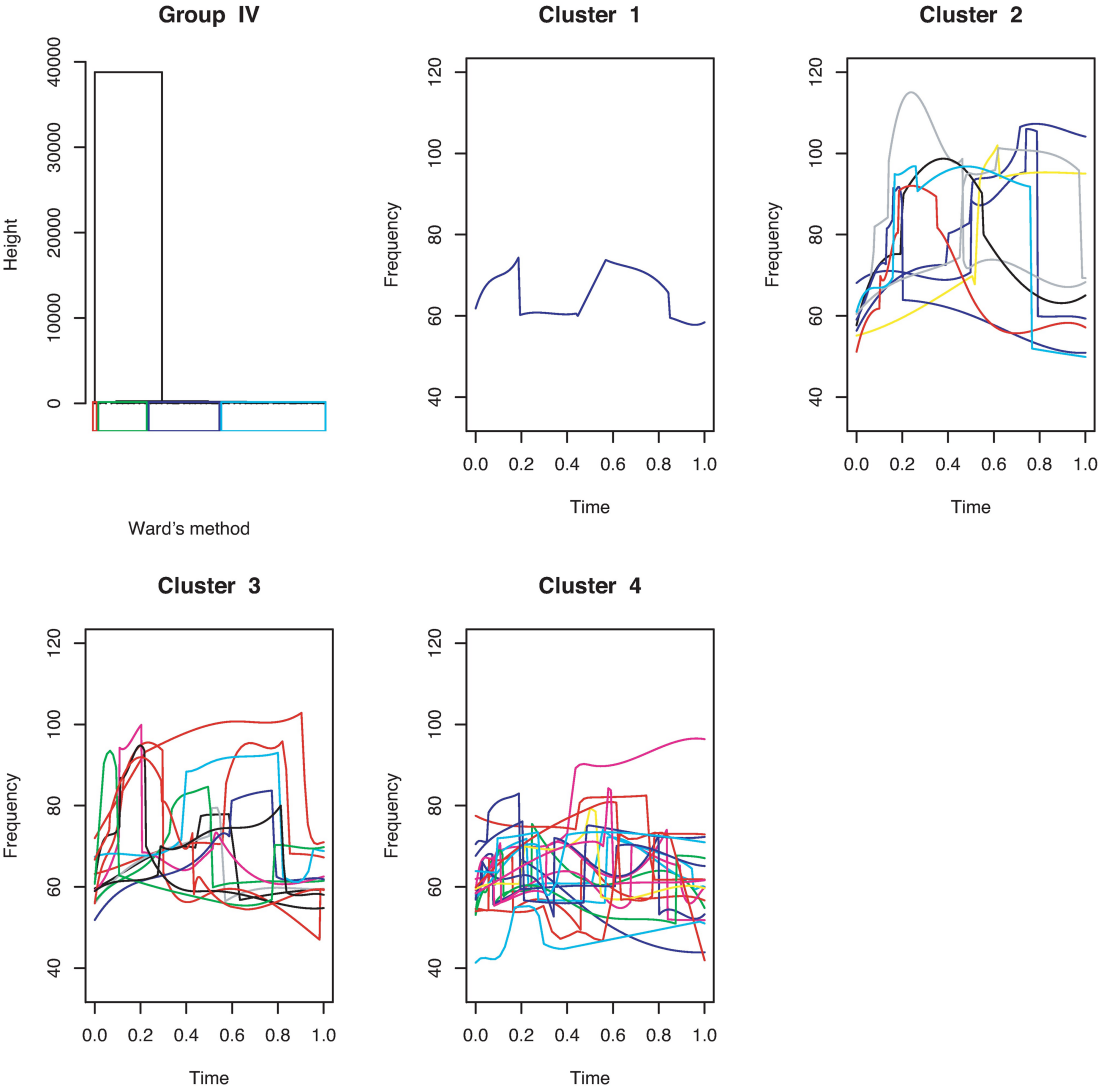
Cluster dendrogram for discontinuous functions with three breakpoints and four clusters.

**Fig 18:**
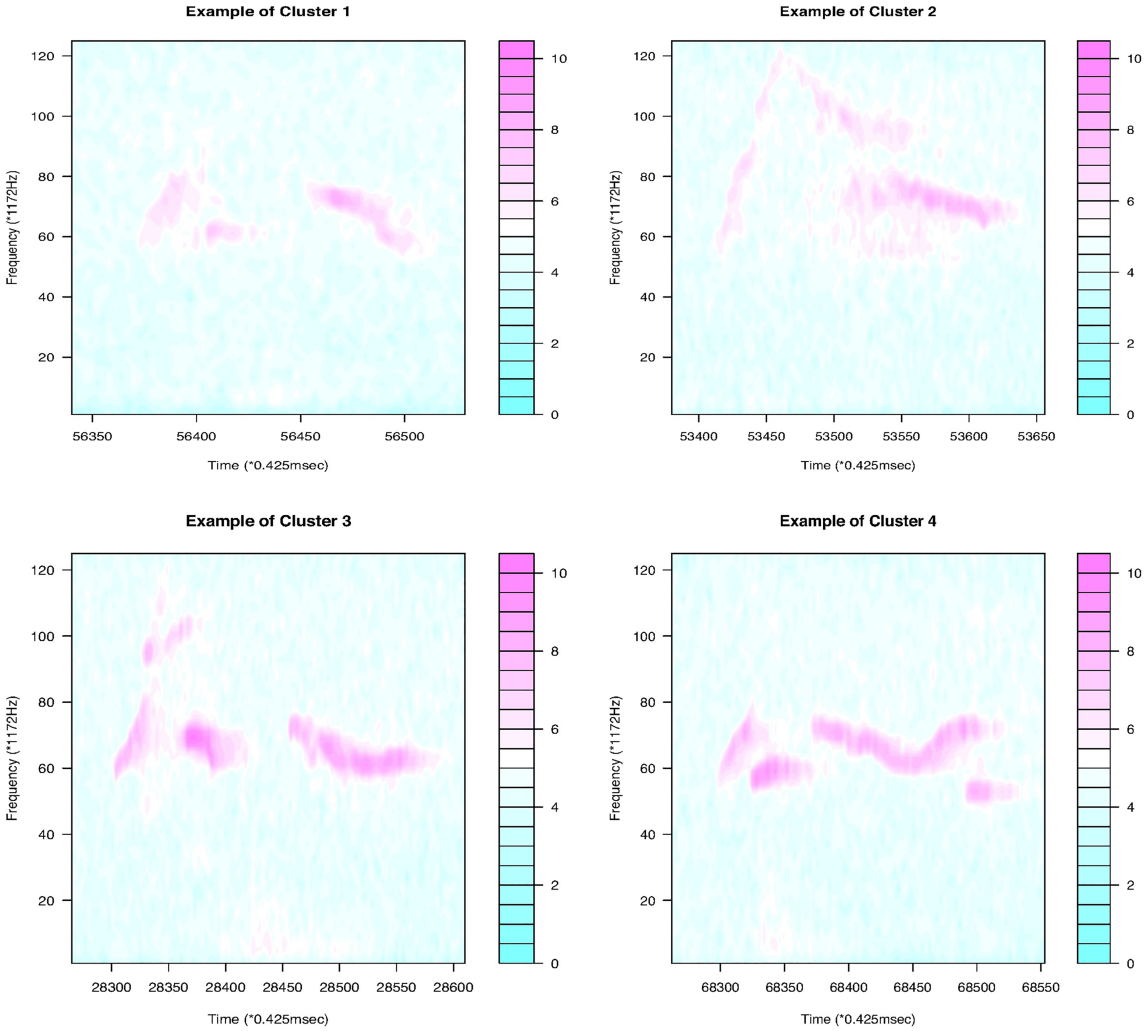
Examples of the four clusters in Group IV.

Fig 11 shows that the continuous USV functions can be split into two clusters (flat or downward, upward). Their examples are given in Fig 12. Fig 13 presents the upward and downward clusters and two outliers of USV curves with one breakpoint (see examples in Fig 14). USV functions with two breakpoints can be separated into four clusters— upward, downward, concave and convex— as shown in Figs 15 and 16. Figs 17 and 18 present dendrograms and four clusters of the USV curves with three breakpoints. We do not separate curves in Group V. Curves in Group V contain four or more breakpoints and have complicated shapes. We have confirmed that most of them are obtained from harmonic USV calls, which should not be characterized by one-dimensional functions.

S3 File of Supporting information shows the groups and clusters of USV functions obtained for mouse C57BL/6JJcl 2396. Among the 225 USV functions in total, 93 are continuous. Most USV calls from mouse C57BL/6JJcl 2420 are harmonic. In this case, modeling USV calls as one-dimensional functions is not appropriate, and jumps on the obtained curves are unstable. If we forcibly analyze the data using the same method, the method works but the result is less reliable. An analysis result is presented in S4 File of Supporting information. In the result, 839 USV functions are obtained. Among them, 270 are continuous.

From the data analysis of the two mouse strains, we also see a difference in the proportions of discontinuous USV functions. The proportions of discontinuous USV functions given by the six mice are as follows.

Because the six mice can be considered independent, the Mann–Whitney test (also called the Wilcoxon rank-sum test) statistic *U* = 0 calculated from Table 3 suggests that with a one-tailed test at a significance level of 0.05 (Statistical Table 8.2 (2), [25]), USV syllables emitted by mice of strain BALB/cAnN have fewer breakpoints than those emitted by C57BL/6JJcl.

**Table 3:** Proportions of discontinuous USV functions in the six datasets.

BALB/cAnN	$\frac{7}{393}$	$\frac{1}{25}$	$\frac{4}{193}$
C57BL/6JJcl	$\frac{259}{374}$	$\frac{132}{225}$	$\frac{569}{839}$

## Discussion

We proposed a functional clustering method for mouse USV data as well as a two-dimensional moving average method and a method for weighting frequency to reduce noise. We defined USV syllables as functions with B-spline basis functions and used multiple knots to define breakpoints for discontinuous USV curves. USV functions with the same number of breakpoints were grouped together and then clustered further by shape.

The methods were proposed for nonharmonic USV calls. It is difficult to use these methods to characterize and classify harmonic USV calls because we define these USV calls as one-dimensional functions. A relatively large number of breakpoints in a USV curve suggests that the curve may be from a harmonic USV call.

In data analysis, we empirically specified some parameters, such as the size of the matrix to use for the moving average, the minimum USV intensity, the quantile for large intensity *p* and the threshold for jump *κ*. The size of the matrix for the moving average was chosen so as to distinguish the USV signals from noise. The minimum of the USV intensity depends on the level of noise and the experimental environment. In our analysis, it was a larger value of *x*_*i*,*j*_ at which there is no USV call. The quantile for large intensity was selected so as to avoid extra jumps in the USV curves. In representing the USV functional data by B-spline basis functions, we proposed the use of more interior knots and a higher breakpoint threshold *κ* for continuous USV calls and the use of fewer interior knots and a lower breakpoint threshold for discontinuous calls. A higher number of interior knots allows the expression of more detailed characteristics of the USV curves and results in good clustering for small differences. However, using fewer interior knots allows characterization of the features of the USV curves simply and the good classification of USV calls with simple shapes. Hence, to cluster USV calls in a broad way, choosing fewer interior knots is better. Finally, the breakpoint threshold *κ* should be chosen by considering the continuity of USV calls and the sizes of all jumps. As we consider frequency jumps in clustering USV functions, identifying the difference in the proportion of discontinuous USV functions is useful in characterizing the USV calls of different mouse strains.

## Supporting information

S1 R Code R module (usv.R) contains code with which to perform the methods described in the article. We provide eight functions in file usv.R. Three of the functions are for reducing noise, defining USV functional data and clustering USV functions by shape. The other five functions are used to plot figures of original USV data, detected USV signals, USV functions, figures for finding the minimum interspace of adjacent USVs and histograms of RMSEs. File usv.R contains code with which to demonstrate the methods and to show the examples and results in the paper.
(R) Click here for additional data file.

S1 Data Mouse USV dataset balb1752.txt. (ZIP)

S2 Data Mouse USV dataset balb1563.txt. (ZIP)

S3 Data Mouse USV dataset balb1565.txt. (ZIP)

S4 Data Mouse USV dataset B6_2358.txt. (ZIP)

S5 Data Mouse USV dataset B6_2396.txt. (BZ2)

S6 Data Mouse USV dataset B6_2420.txt. (BZ2)

S1 File Analysis result of dataset balb1563.txt. (PDF)

S2 File Analysis result of dataset balb1565.txt. (PDF)

S3 File Analysis result of dataset B6_2396.txt. (PDF)

S4 File Analysis result of dataset B6_2420.txt. (PDF)
