# Supplementary material for: Functional clustering of mouse ultrasonic vocalization data
Source: PLoS One. 2018 May 9;13(5):e0196834. doi: 10.1371/journal.pone.0196834 (PMC5942836; doi:10.1371/journal.pone.0196834)
Supplement: S1 File — (PDF) [file pone.0196834.s008.pdf]

Supporting Information for
"Functional clustering of mouse ultrasonic vocalization data"
by Dou et al.
— Analysis result of dataset `balb1563.txt`

From data of mouse BALB/cAnN 1563, we detected 25 USV calls, among which 24 are continuous and one is discontinuous.

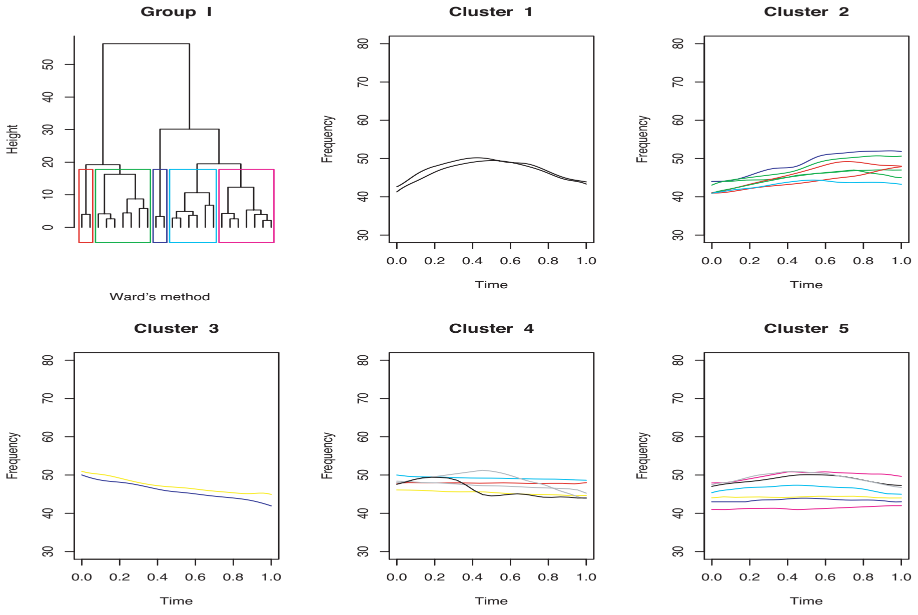

Figure 1: Cluster dendrogram and clustering of continuous USV functions from mouse BALB/cAnN 1563.

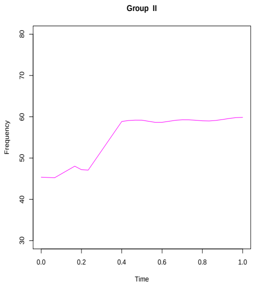

Figure 2: Cluster dendrogram and clustering of USV functions with one breakpoint from mouse BALB/cAnN 1563.
