# Supplementary material for: Functional clustering of mouse ultrasonic vocalization data
Source: PLoS One. 2018 May 9;13(5):e0196834. doi: 10.1371/journal.pone.0196834 (PMC5942836; doi:10.1371/journal.pone.0196834)
Supplement: S2 File — (PDF) [file pone.0196834.s009.pdf]

Supporting Information for “Functional clustering of mouse ultrasonic vocalization data” by Dou et al. — Analysis result of dataset `balb1565.txt`

From the data of mouse BALB/cAnN 1565, we obtain 189 continuous USV calls and four discontinuous USV calls.

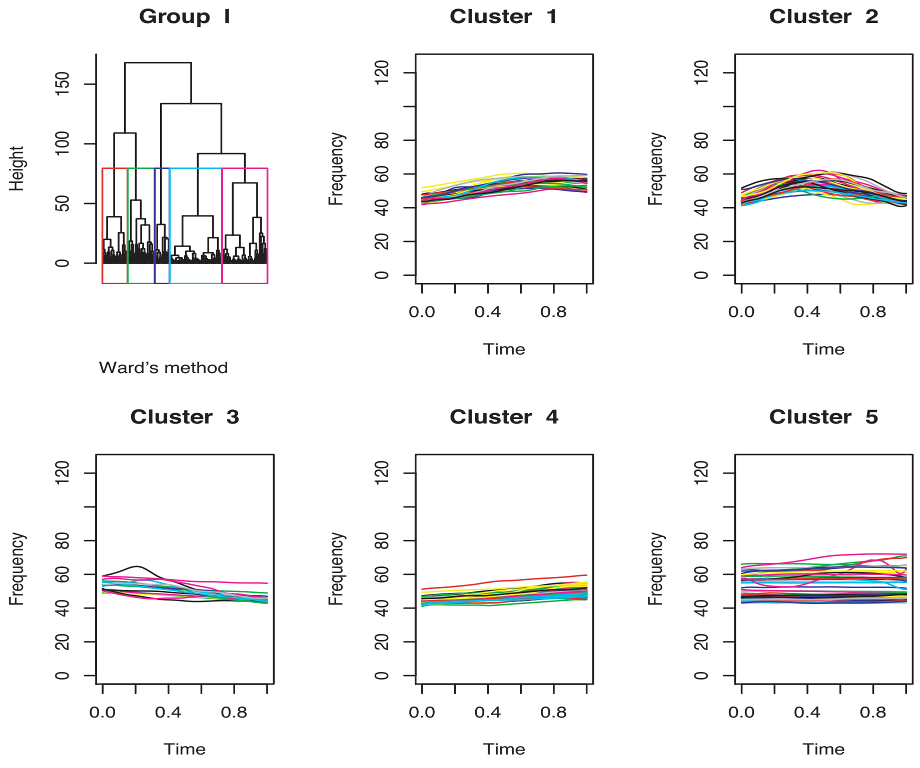

Figure 1: Cluster dendrogram and clustering of continuous USV functions from mouse BALB/cAnN 1565.

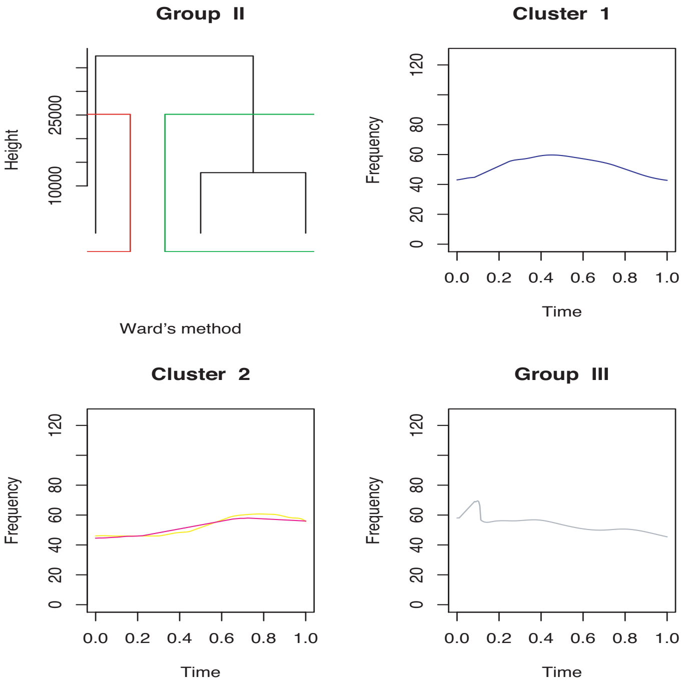

Figure 2: Cluster dendrogram and clustering of discontinuous USV functions from mouse BALB/cAnN 1565.
