# Supplementary material for: Functional clustering of mouse ultrasonic vocalization data
Source: PLoS One. 2018 May 9;13(5):e0196834. doi: 10.1371/journal.pone.0196834 (PMC5942836; doi:10.1371/journal.pone.0196834)
Supplement: S3 File — (PDF) [file pone.0196834.s010.pdf]

Supporting Information for
"Functional clustering of mouse ultrasonic vocalization data"
by Dou et al.
— Analysis result of dataset `B6_2396.txt`

The groups and clusters of USV functions obtained for mouse C57BL/6JJcl 2396 are obtained as follows. Among the 225 USV functions in total, 93 continuous functions can be split into four clusters: flat with lower frequency, upward, flat with higher frequency and downward. Two clusters (jump-up and jump-down) share the USV functions with one breakpoint. Curves with two and three jumps are also clustered.

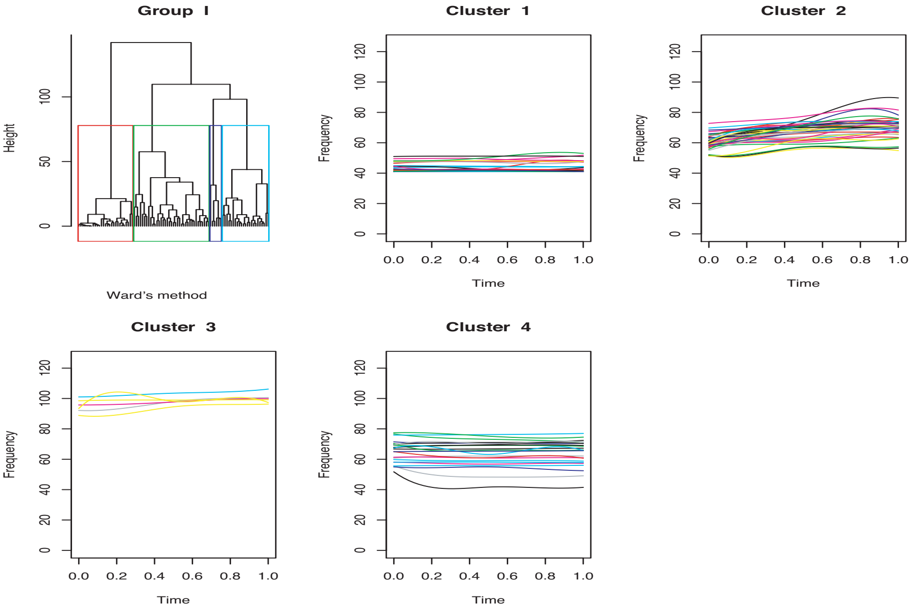

Figure 1: Cluster dendrogram and clustering of continuous USV functions for mouse C57BL/6JJcl 2396.

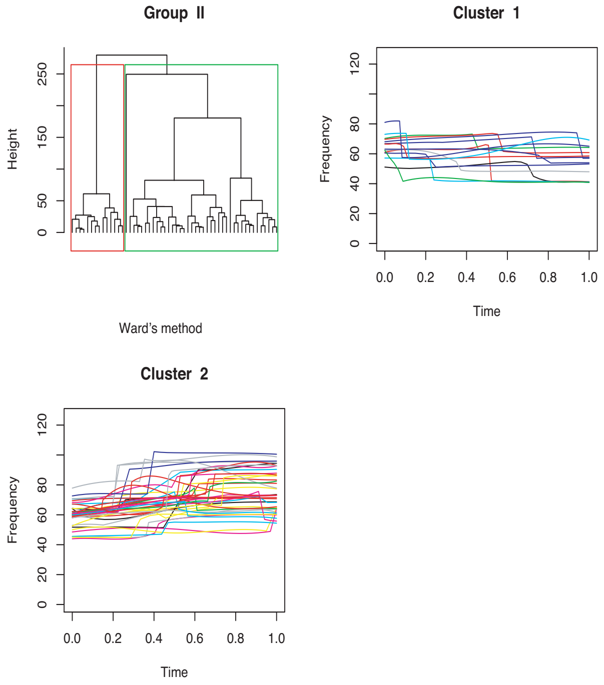

Figure 2: Cluster dendrogram and clustering of discontinuous USV functions with one breakpoint for mouse C57BL/6JJcl 2396.

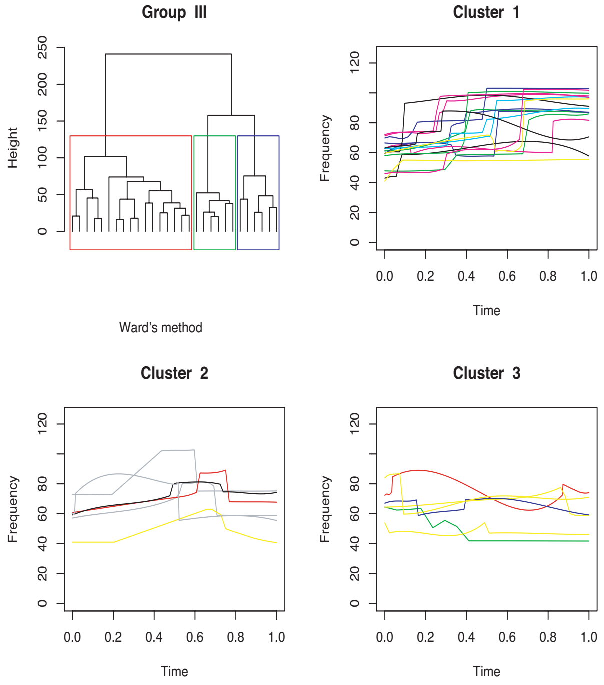

Figure 3: Cluster dendrogram and clustering of discontinuous USV functions with two breakpoints for mouse C57BL/6JJcl 2396.

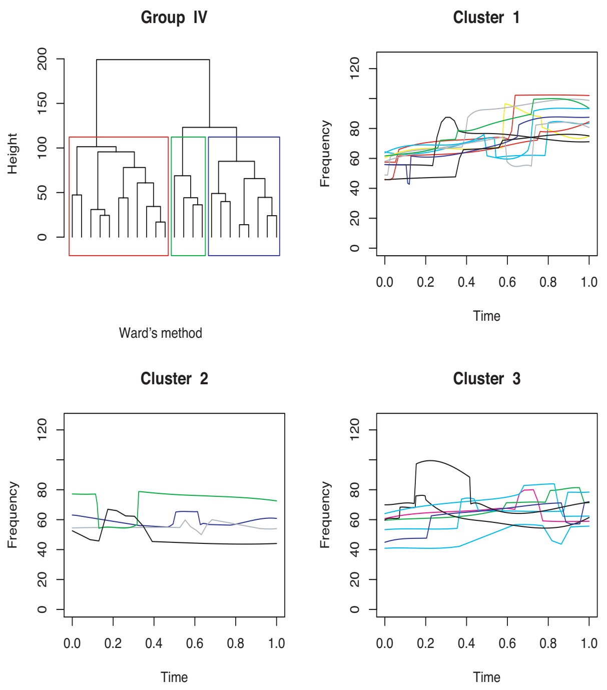

Figure 4: Cluster dendrogram and clustering of discontinuous USV functions with three breakpoints for mouse C57BL/6JJcl 2396.
