# Supplementary material for: Functional clustering of mouse ultrasonic vocalization data
Source: PLoS One. 2018 May 9;13(5):e0196834. doi: 10.1371/journal.pone.0196834 (PMC5942836; doi:10.1371/journal.pone.0196834)
Supplement: S4 File — (PDF) [file pone.0196834.s011.pdf]

Supporting Information for
"Functional clustering of mouse ultrasonic vocalization data"
by Dou et al.
— Analysis result of dataset `B6_2420.txt`

Most USV calls from mouse C57BL/6JJcl 2420 are harmonic. In this case, modeling USV calls as one-dimensional functions is not appropriate, and jumps on the obtained curves are unstable. If we forcibly analyze the data using the same method, the method works but the result is less reliable. The following is a clustering result.

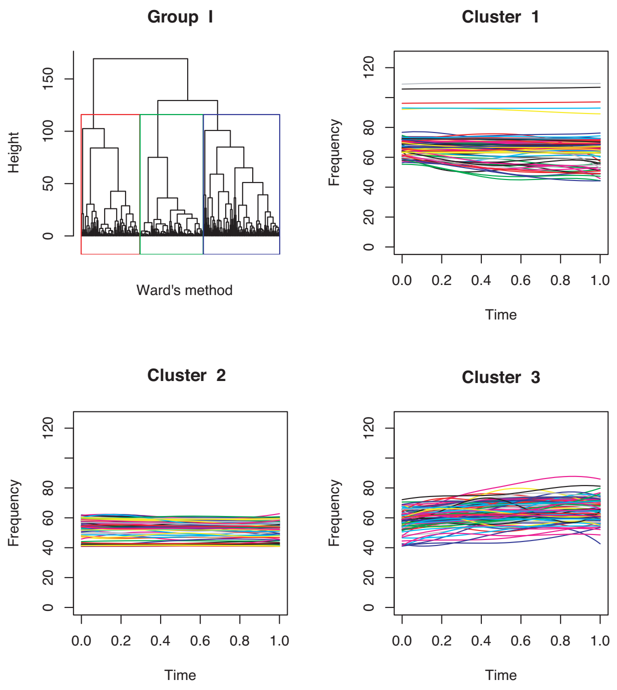

Figure 1: Cluster dendrogram and clustering of continuous USV functions from mouse C57BL/6JJcl 2420.

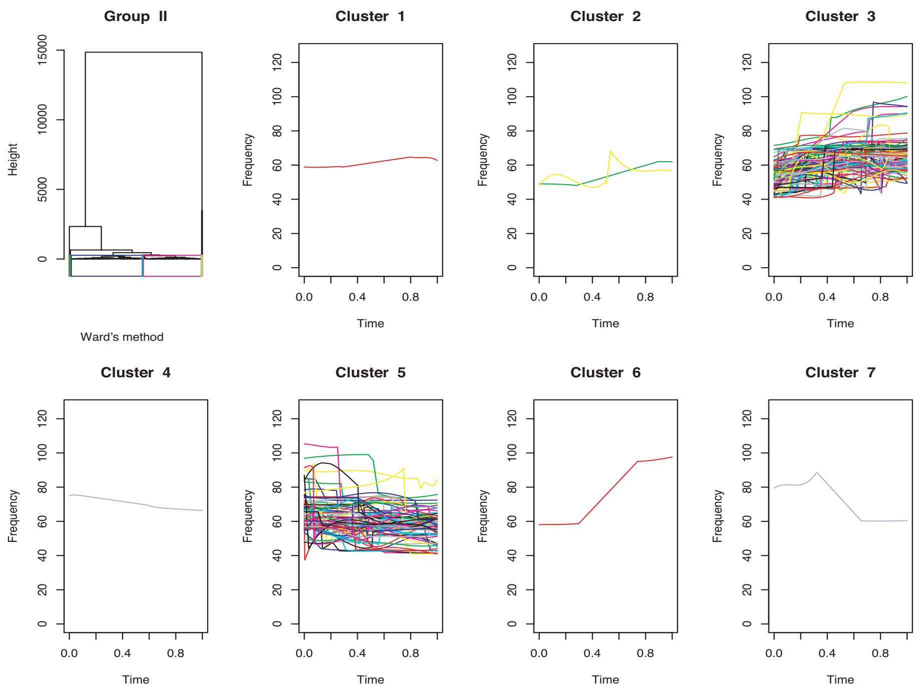

Figure 2: Cluster dendrogram and clustering of USV functions with one breakpoint from mouse C57BL/6JJcl 2420.

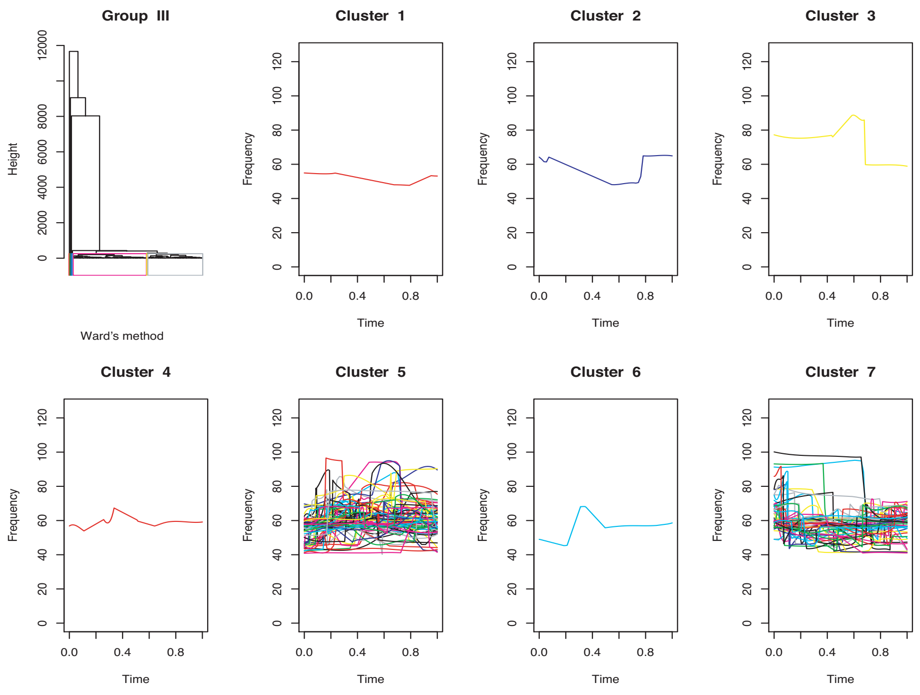

Figure 3: Cluster dendrogram and clustering of USV functions with two breakpoints from mouse C57BL/6JJcl 2420.

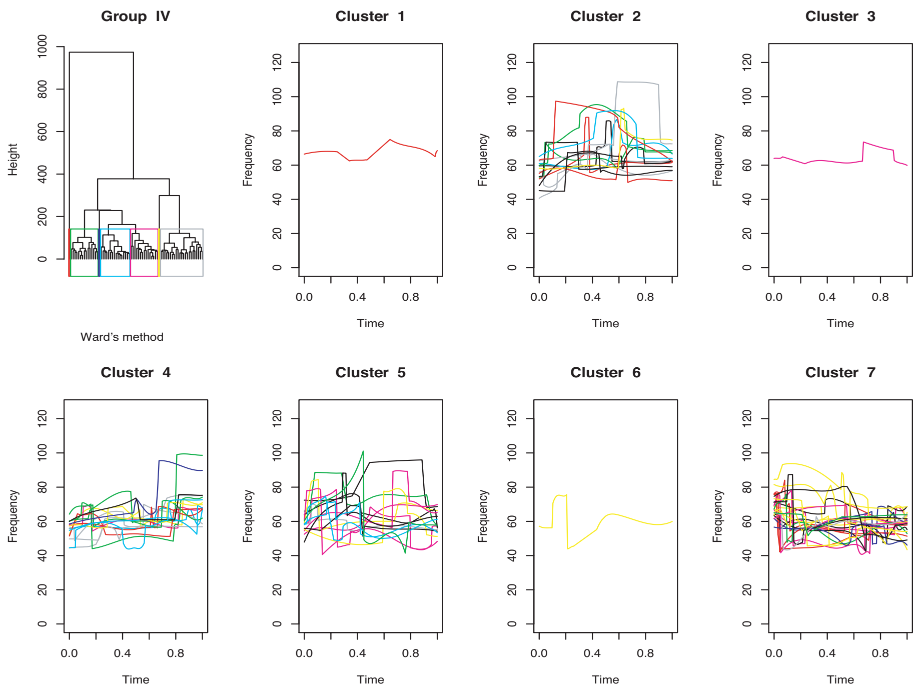

Figure 4: Cluster dendrogram and clustering of USV functions with three breakpoints from mouse C57BL/6JJcl 2420.
